# Application of Deep Learning Technology in Strength Training of Football Players and Field Line Detection of Football Robots

**DOI:** 10.3389/fnbot.2022.867028

**Published:** 2022-06-29

**Authors:** Daliang Zhou, Gang Chen, Fei Xu

**Affiliations:** ^1^School of PE, Nanjing Xiaozhuang University, Nanjing, China; ^2^School of Physical Education, Hangzhou Normal University, Hangzhou, China

**Keywords:** deep learning, human action recognition, dual-stream network, functional strength training, recognition accuracy

## Abstract

The purpose of the study is to improve the performance of intelligent football training. Based on deep learning (DL), the training of football players and detection of football robots are analyzed. First, the research status of the training of football players and football robots is introduced, and the basic structure of the neuron model and convolutional neural network (CNN) and the mainstream framework of DL are mainly expounded. Second, combined with the spatial stream network, a CNN-based action recognition system is constructed in the context of artificial intelligence (AI). Finally, by the football robot, a field line detection model based on a fully convolutional network (FCN) is proposed, and the effective applicability of the system is evaluated. The results demonstrate that the recognition effect of the dual-stream network is the best, reaching 92.8%. The recognition rate of the timestream network is lower than that of the dual-stream network, and the maximum recognition rate is 88%. The spatial stream network has the lowest recognition rate of 86.5%. The processing power of the four different algorithms on the dataset is stronger than that of the ordinary video set. The recognition rate of the time-segmented dual-stream fusion network is the highest, which is second only to the designed network. The recognition rate of the basic dual-stream network is 88.6%, and the recognition rate of the 3D CNN is the lowest, which is 86.2%. Under the intelligent training system, the recognition accuracy rates of jumping, kicking, grabbing, and starting actions range to 97.6, 94.5, 92.5, and 89.8% respectively, which are slightly lower than other actions. The recognition accuracy rate of passing action is 91.3%, and the maximum upgrade rate of intelligent training is 25.7%. The pixel accuracy of the improved field line detection of the model and the mean intersection over union (MIoU) are both improved by 5%. Intelligent training systems and the field line detection of football robots are more feasible. The research provides a reference for the development of AI in the field of sports training.

## Introduction

Football has become the most popular sport in the world today due to its strong antagonism and enjoyment, and it is known as “the largest sport in the world.” As the top football league, the Fédération Internationale de Football Association (FIFA) World Cup is held every 4 years. As of the 21st World Cup in 2018, the global TV audience has reached more than 3.5 billion (Pons et al., 2021). Football requires high physical fitness of athletes, especially strong jumping ability, which is an indispensable skill for shooting. It not only requires athletes' leg strength, but also other parts of the body (Bueno et al., 2021). The functional training of football refers to the realization of specific sports functions of the body for football (Teixeira et al., 2021). In addition to jumping ability, functional strength training in football sports includes training for kicking strength of their legs, the stability of athletes' upper and lower limbs during football competition, and training for athletes' acceleration and starting strength during competitions, etc. (Vella et al., 2021). Currently, the functional strength training of football players requires the coaches to adjust training items, supervise the athletes to complete strength training, and to analyze the training situation (Guan and Wang, 2021). Due to the limitation of the number of coaches and the interference factors of man-made supervision, the speed and strength of the athletes are improved to a small extent within a certain period. Therefore, artificial intelligence (AI) is introduced to study the functional training of football. Since the requirements for AI in the functional training of football players are in semantic extraction and action recognition, based on deep learning (DL) and human action recognition related knowledge, the speed and strength improvement of athletes in functional training is studied. Currently, human action recognition is one of the research hotspots in the field of computer vision, and it is gradually being applied in many fields, such as virtual reality, video surveillance, the interaction of AI, medical assistance, etc. (Materne et al., 2020). The research content of human action recognition mainly includes the extraction and analysis of human action and the detection of moving targets (Parim et al., 2021). The research often encounters discrepancies in different environments, so many methods of expressing features have been proposed, such as DL algorithms. DL algorithms are different from traditional artificial feature extraction, which saves a lot of time and manpower (Aslam et al., 2021).

Deep learning algorithms belong to the field of machine learning and are mainly used in the learning of complex semantics by machines. The DL uses hierarchical extraction of features and feature learning methods, and currently, it is widely used in audio recognition, processing of natural language, and in other fields (Khan et al., 2021). As one of the common models of DL, the convolutional neural network (CNN) shows great advantages in visual recognition tasks. Human action recognition will be studied by combining CNN with the DL algorithm (Khan et al., 2020). Human action recognition under DL mainly extracts the global and local information of the video image in the computer, and it performs feature extracting and analysis on this part of the information, and then conducts the recognition of the target behavior (Ullah et al., 2020).

As an important experimental platform for the research of AI application technology and multi-agent system (MAS), the football robot is a vital way to break through the research in the field of AI technology. Meanwhile, it is also extremely challenging for scientific researchers. Its related technologies include robotics, AI, intelligent control technology, sensor technology, communication, computer and image processing, and other fields. A football robot is a typical MAS in the form of competition. The research on its structure theory and control method has promoted the development of methods and theories of robotics and AI technology (Setyawan et al., 2022). Intelligent robots have been widely used in various fields of social life. As one of the intelligent robots, football robot games have often been held around the world in recent years. It integrates technology, viewing and fun, effectively stimulates the enthusiasm of young people for research, and provides a stage for cultivating all aspects of ability. Students closely combine scientific knowledge with practice, link theory with practice, and exert their intelligence through practical operation, teamwork, innovation, creation, etc. (Suwarno et al., 2022). The actual technological level and control skills can be evaluated in the fairest, most reasonable, and objective way through the robot football game. Through the competition, its own deficiencies are found, to improve and innovate technology, and promote the development of related technologies (Wang, 2022). The Robo Cup Humanoid Competition is the highest-level competition in the field of humanoid football robots. The robot captures the field image through the camera, performs field line detection on the image, extracts the feature information in the field line that can be used for robot positioning, combines with the robot's movement information, performs robot self-positioning, and makes decisions and path planning based on the detection and positioning information to complete match (Watanabe et al., 2022). Since the current football robot positioning detection is unstable, it will be combined with the fully convolutional network (FCN) for research.

The traditional functional strength training of football players has the problems of insufficient coaches and slow speed in improving the speed and strength of football players. In the era of AI, the training problem of football players can be effectively solved. Based on the current situation of football player training, the AI is introduced to study the players' functional training, aiming to improve the detection technology of football robots. First, the research background of football training and football robots in the era of AI is introduced. Second, combined with video recognition technology, a functional strength training system by AI and CNN is established, combining time flow and spatial flow network to build a field line positioning system for football robots based on FCN. Finally, the feasibility of the two systems is evaluated. The innovation lies in the application of the human action recognition system under DL to solve the problems existing in the ability training of football players. The main difference between the designed algorithm and other algorithms is to propose an improved model based on FCN and residual network for field line detection of football robots. The used data set is an image data set containing 4,800 videos. To sum up, the feasibility of the provided intelligent training system and the high accuracy of the football detection of football robots are obtained. The research results provide a new way for the development of strength training of football players and the detection technology of football robots.

## Design of an Intelligent System for Functional Strength Training and Research on Field Line Detection of Football Robot

### Research Status of Human Action Recognition

Human action recognition algorithms before the emergence of DL algorithms were mainly studied through shallow learning and spatiotemporal features. For example, sampling the pixel points in the video for dense trajectory acquisition, and extracting feature information as the result of the recognition action; acquiring the research target trajectory in the video by the information of the optical flow field, extracting feature information, and then performing feature encoding to obtain recognition results; researching the key point of optical flow information in the video through the sequence of video; combining Gaussian distribution to perform feature extraction on the information in the video, etc. (Fan et al., 2020).

Since the system of human action recognition involves a lot of domains, including feature selection, machine vision, pattern recognition, etc., it is also a very challenging processing method for the computer (Pareek and Thakkar, 2020), so it has important significance in the research of human action recognition. The current research on human action recognition has made great progress, but there are still some shortcomings, such as insensitivity to the differences among different targets, difficulty in processing under the influence of complex video environment background, and differences between video databases and real data, etc. (Ozcan and Basturk, 2020). The emergence of DL algorithms has gradually replaced artificial representation methods, and the above problems have been studied. Methods of action recognition based on DL include CNN and dual-stream algorithms (Abdelbaky and Aly, 2021).

The main research direction in DL is the dual-stream algorithm, which aims at using the two dimensions of space and time to study video, and then combines the optical flow image and the video frame image to train the DL model, and to finally obtain the recognition result (Lagemann et al., 2021). The CNN has a simple network structure and a large-scale processing data set, so it is widely used. Among them, the 3D CNN extracts and recognizes video images through the three-dimensional convolution kernel, which is more comprehensive than the two-dimensional algorithm (Fan et al., 2020).

The research on human action recognition has been carried out earlier, but it has only been applied in sports in recent years. Research on human movement in the field of sports involves sports, such as basketball, badminton, etc. Football research is mainly focused on tracking the trajectory of football players in the game, and there is less research on strength training (Stoeve et al., 2021). Thakkar and Shah (2021) proposed the use of wearable devices for action recognition (Thakkar and Shah, 2021). In addition, the research methods for human action recognition include computer vision research (Newman et al., 2021). Football is a highly antagonistic sport and is not suitable for wearing equipment. Therefore, the visual aspects of human action recognition are analyzed.

### Research Status of Football Robots

In the 1992 International AI Conference, Professor Alan Mackworth of Canada first proposed the idea of robot football in his paper, “On Seeing Robot.” The Federation of International Robot Soccer Association (FIRA) competition was first proposed by Professor Kim Jong-hwan of South Korea in 1995. The first and second international competitions were held in Korea in 1996 and 1997, respectively (Chen and Gao, 2020). In June 1997, the FIRA was announced during the second micro-robot football competition. Since then, FIRA holds an international competition every year and an academic conference (FIRA Congress) to exchange experience and technology in the robot football research. In 2011, the 16th FIRA Robo World Cup and Congress were held in Taiwan for the first time, with more than 10 national teams including South Korea, Canada, Mexico, the United Kingdom, Singapore, Malaysia, Thailand, Argentina, Slovakia, and China participated in the competition, and 53 teams competed on the same stage. This is the FIRA game, one of the international robot soccer game series organized by FIRA so far (Chen, 2020).

With the development of the FIRA competition, another major series of football robot competitions, the Robo Cup, has also been developed by leaps and bounds during the same period. The Robo Cup J League was planned in June 1993 by Minoru Asada, Hiroaki Kitano, and Yasuo Kuniyoshi. However, within a month, it received a positive response from scientific researchers around the world, and therefore changed its name to Robo Cup, that is, the Robot Football World Cup. In 1997, the first official competition and meeting of the Robo Cup was held in Nagoya, Japan, with 38 teams from 11 countries participating in the competition (Hong et al., 2021). The competition was a huge success. Before this competition, many scientific researchers did a lot of pre-competition preparations, such as drafting rules, organizing simulation group competitions, etc., to ensure the success of this competition. Since then, the Robo Cup competition is held around the world every year, and then the exchange meeting on its scientific research progress is held, which provides a good communication platform for scientific researchers and promotes the rapid development of their related scientific and technological fields (Houtman et al., 2021).

In 1997, Chinese researchers began to study robot football, starting relatively late. In 1998, the Chinese branch of the FIRA was established. In the Robo Cup held in Brazil in August 1999, the New Neu team of Northeastern University won the first place in standard action and the fifth place in Mriosot, realizing the breakthrough of the Chinese team's 0 gold medal in the Robo Cup (Antonioni et al., 2021). In 2000, the Robo Cup was held in Beijing for the first time. In the Robo Cup in August 2001, 8 championships among 9 competitions were won by the Chinese team, which fully proves that China has achieved a very high development level in the field of robot football research. After more than 10 years of unremitting efforts, with the strong support of FIRA and the Chinese Society for AI, the Chinese robot football team has spread all over the country. So far, there are more than 200 teams belonging to more than 80 universities. More and more scientific research institutes and universities have begun to organize scientific research forces to join the team of football robots, which has greatly promoted the development of football robots and the related fields of science and technology.

The rules of the game before 2013 stipulated that the game ball is orange, the goal is yellow or blue, and the field line is white, so the target detection algorithms are all algorithms based on color segmentation. As the rules of the game continue to change, the ball becomes a mixture of black and white, the goal and field lines are both white, and the algorithm based on color segmentation is no longer effective. Based on the odometry-based positioning method, Park et al. (2022) calculated the current robot's pose relative to the initial moment by accumulating the measurement results (Park et al., 2022). To achieve higher accuracy, filtering methods, such as Kalman filtering can be used for coordination. The advantage of this method is that it can quickly provide the robot pose, but the disadvantage is that it has accumulated errors. As the movement time or distance increases, the error of its pose estimation will also increase. Due to the high confrontation of robot football games, the robot collisions between them occur frequently, so this method is only suitable for short-term and short-distance self-positioning. Jeong et al. (2022) proposed a positioning method based on sensors, such as laser rangefinders and sonars, using a rotating mirror mechanism to emit laser beams outwards, and to detect the laser beams reflected by objects, and to obtain external environmental information. This positioning method has high positioning accuracy, good anti-interference, no cumulative error, and a short positioning period, but the equipment is expensive, and the cost is high (Jeong et al., 2022). Colombini et al. (2022) proposed a vision-based positioning method by obtaining images of surrounding scenes through visual sensors, using some natural or artificial features in the scenery, and obtaining models of the surrounding environment through image processing methods to achieve positioning (Colombini et al., 2022). According to the different positioning markers, this method can be divided into the positioning method based on road signs and based on white lines. Due to the limited observation range of general omnidirectional vision, it is difficult to observe enough positioning marks in most areas of the site. The CNN has developed rapidly in recent years. Its excellent performance in image processing has led more and more researchers to abandon algorithms based on color segmentation and use CNN to detect balls and goals but still uses a color segmentation-based algorithm to detect yield lines. The field line detection algorithm based on color segmentation has a small amount of calculation and a fast operation speed, but it is only suitable for the situation of stable lighting conditions, and the detection effect becomes worse when the lighting conditions change. The technical committee of the Robo Cup has issued a notice that the competition venue will be illuminated with natural light. Therefore, the field line detection effect based on the color segmentation algorithm will become unstable, and new methods need to be studied to cope with changes in lighting conditions and improve the detection stability.

Therefore, in the field detection part of the football robot, aiming at improving the detection stability, based on the FCN model, an improved model is proposed for the field line detection of the football robot. The design idea of the residual network model is adopted, and the number of network convolutional layers is increased. The residual structure is introduced to enhance the ability of the network to extract image features, and more low-level field line information is incorporated in the process of upsampling to improve the accuracy of the field site line.

### Theory Basis of DL

#### The Neuron Model of Neural Network

The principle of DL is to improve the computer performance from both new data and historical experience after being input into the computer (Sarker, 2021). The research of DL algorithms originated from the development of artificial neural networks, so DL also has the characteristics of neural network hierarchical structure (Chen et al., 2021). The neural network model is a model similar to the neural mechanism of the human brain designed by humans in order to allow computers to recognize behaviors and understand images and other complex behaviors. The neural model contains a hierarchical structure composed of neuron nodes. Neurons are its basic components. The specific expression of neurons is shown in Equation (1) (Kuwana et al., 2020).


(1) $$\begin{array}{l} \sigma_{w,b}\left(X\right)=\sigma \left(W^{T}x\right)=\sigma \left(\overset{n}{\underset{i=0}{\sum }}w_{i}x_{i}+b\right) \end{array}$$

In Equation (1), *X* is the output value; *W* is the weight; b means the offset value; *n* is the number of inputs, *i* represents the input sequence number; σ is the output value.

Neurons between different levels are connected through input and output, and the relationship between output and input is described by an activation function. In general action recognition research, choosing a non-linear function as the activation function is of great significance in solving complex problems in real life. The more commonly used activation functions include the hyperbolic tangent function, the step function, the S-shaped growth curve, modified linear unit, etc. The specific expressions of these four functions are shown in Equations (2)–(5).


(2) $$\begin{array}{l} \tanh\left(x\right)=\frac{e^{x}-e^{-x}}{e^{x}+e^{-x}} \end{array}$$

Equation (2) is the expression of the hyperbolic tangent function, and the output value ranges from −1 to 1.


(3) $$\begin{array}{l} sgn\left(x\right)=\{\begin{array}{c} 1,\;x\geq 0\\ 0,\;x<0 \end{array} \end{array}$$

Equation (3) is the expression of the step function. It can be seen from Equation (3) that when the input value is less than 0, the output of the step function is 0, which means that the neuron has not received excitation; when the input value is greater than or equal to 0, the output of step function is 1. It can be seen that the step function is an idealized expression, and it is used to solve complex practical problems.


(4) $$\begin{array}{l} sigmoid\left(x\right)=\frac{1}{1+e^{-x}} \end{array}$$

Equation (4) is the expression of the S-shaped growth curve function, and the output value range is between 0 and1. When the input value of the S-shaped growth curve function has an extreme value, the gradient of the function approaches 0, which will affect the back propagation, so it is not suitable for DL.


(5) $$\begin{array}{l} Relu\left(x\right)=max\left(0,\;x\right) \end{array}$$

Equation (5) is the expression of the modified linear element function. The calculation of the modified linear element is relatively simple, and it has the characteristics of fast derivative convergence, so it is widely used to study the problem of gradient disappearance.

**Figure 1:**
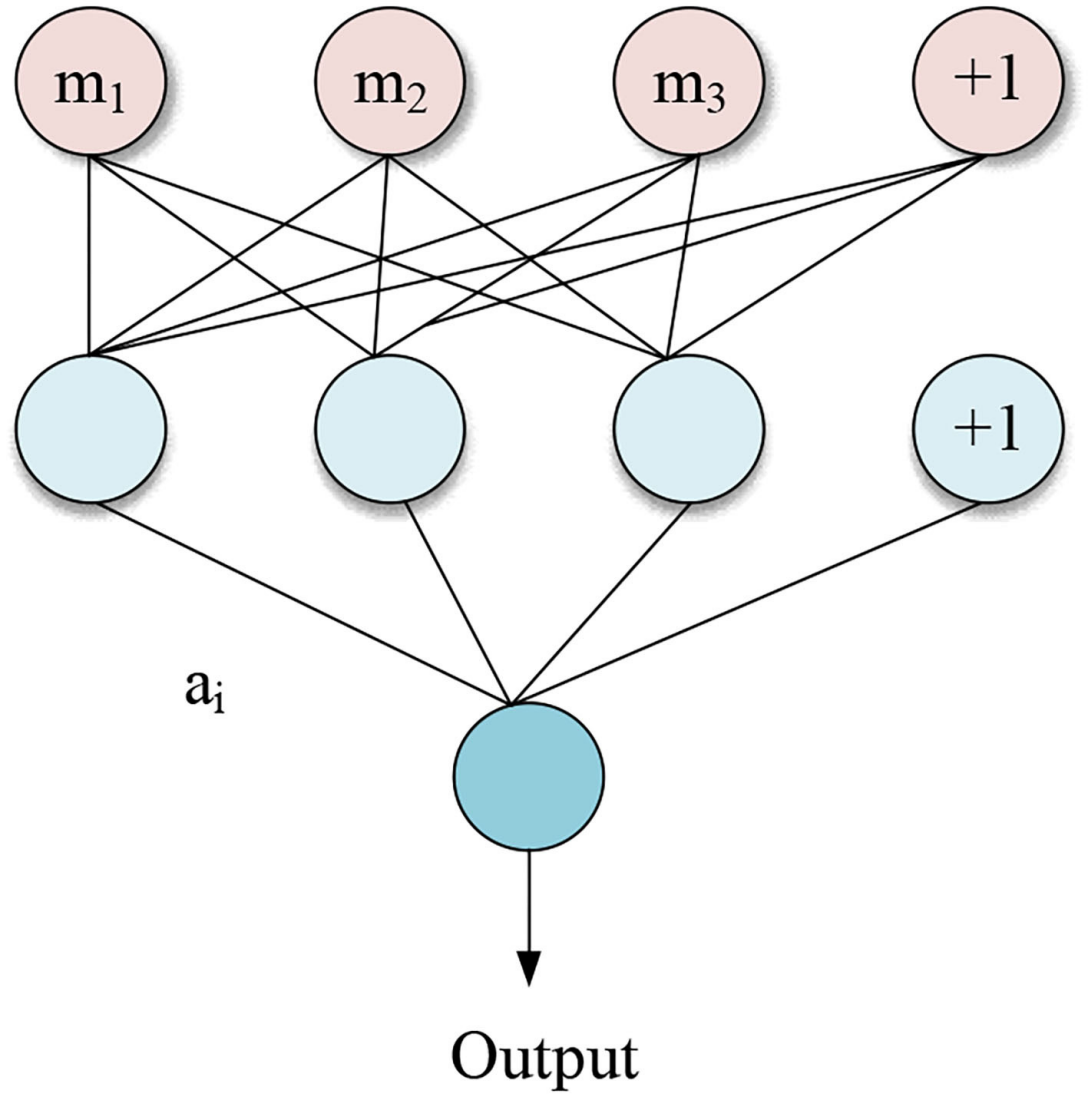
Structure of feedforward neural network.

The feedforward neural network composed of a certain combination of multiple neurons is an important part of the study. The function of the feedforward neural network is to spread samples. The network structure of the feedforward neural network has no closed loops and no feedback information in the forward direction. The specific structure is shown in Figure 1.

Figure 1 shows the three components of the feedforward neural network, including the output layer, input layer, and hidden layer. In the figure, a_i_ represents the output of the *i*th neuron, and the specific equation expressions are shown in Equations (6)–(9) (Haldorai and Ramu, 2020).


(6) $$\begin{array}{l} a_{1}^{\left(2\right)}=f\left(H_{11}^{\left(1\right)}m_{1}+H_{12}^{\left(1\right)}m_{2}+H_{13}^{\left(1\right)}m_{3}+c_{1}^{\left(1\right)}\right) \end{array}$$

(7) $$\begin{array}{l} a_{2}^{\left(2\right)}=f\left(H_{21}^{\left(1\right)}m_{1}+H_{22}^{\left(1\right)}m_{2}+H_{23}^{\left(1\right)}m_{3}+c_{2}^{\left(1\right)}\right) \end{array}$$

(8) $$\begin{array}{l} a_{3}^{\left(2\right)}=f\left(H_{31}^{\left(1\right)}m_{1}+H_{32}^{\left(1\right)}m_{2}+H_{33}^{\left(1\right)}m_{3}+c_{3}^{\left(1\right)}\right) \end{array}$$

(9) $$\begin{array}{l} q_{H,c}\left(m\right)=a_{1}^{\left(3\right)}=f\left(H_{11}^{\left(2\right)}a_{1}^{\left(2\right)}+H_{12}^{\left(2\right)}a_{2}^{\left(2\right)}+H_{13}^{\left(2\right)}a_{3}^{\left(2\right)}+c_{1}^{\left(2\right)}\right) \end{array}$$

In Equations (6)–(9), *m* is the input information; *H* is the weight parameter; *c* is the bias parameter; *q* is the final output result of the hidden layer, and ${\text{a}}_{i}^{\left(1\right)}$ represents the output of *i*th neuron node in the *l*th layer. ${\text{H}}_{ij}^{\left(l\right)}$ represents the weight of the *i*th node of the *l*th layer connected to the *i*th node of the first layer, and ${\text{c}}_{i}^{\left(l\right)}$ represents the bias parameter of the *i*th neuron of layer *l*, and *f* is the activation function.

The input weighted sum is introduced to represent the *i*th node of the *l*th layer, and the above equations are simplified to obtain Equations (10) and (11).


(10) $$\begin{array}{l} v^{l}=H^{\left(l-1\right)}f\left(v^{\left(1-l\right)}\right)+c^{\left(l-1\right)} \end{array}$$

(11) $$\begin{array}{l} a^{l}=f\left(v^{\left(l\right)}\right) \end{array}$$

*f* is the excitation function in Equations (10) and (11).

Next, data update is needed to complete network training. The update of parameter is performed by the back propagation method. The loss function expression of the network output layer is shown in Equation (12).


(12) $$\begin{array}{l} P\left(H,c;m,y\right)=\frac{1}{2}{\left\|q_{H,c}\left(m\right)-y\right\|}^{2} \end{array}$$

In Equation (12), *y* is the output result obtained, and *P* is the loss function.

The residuals of different nodes are expressed by Equation (13).


(13) $$\begin{array}{l} \beta_{i}^{\left(l\right)}=\left(\overset{s_{l+1}}{\underset{j=0}{\sum }}H_{ji}^{\left(l\right)}\beta_{i}^{\left(l+1\right)}\right)f^{\prime }\left(v_{i}^{\left(l\right)}\right) \end{array}$$

In Equation (13), *s* represents the number of nodes in all layers.

The relationship between the loss function and the input value is expressed as Equations (14) and (15) using residuals.


(14) $$\begin{array}{l} \frac{\partial }{\partial H_{ji}^{\left(l\right)}}P\left(H,c;m,y\right)=a_{j}^{\left(l\right)}\beta_{i}^{\left(l+1\right)} \end{array}$$

(15) $$\begin{array}{l} \frac{\partial }{\partial b_{j}^{\left(l\right)}}P\left(H,c;m,y\right)=\beta_{i}^{\left(l+1\right)} \end{array}$$

Equations (14) and (15) express the rate of change of the loss function to the input.

Finally, the gradient descent method is used to update the parameters *H* and *c*, and equations (16) and (17) are obtained.


(16) $$\begin{array}{l} H_{ji}^{\left(l\right)}=H_{ji}^{\left(l\right)}-\alpha \frac{\partial }{\partial H_{ji}^{\left(l\right)}}P\left(H,c\right) \end{array}$$

(17) $$\begin{array}{l} c_{j}^{\left(l\right)}=c_{j}^{\left(l\right)}-\alpha \frac{\partial }{\partial c_{j}^{\left(l\right)}}P\left(H,c\right) \end{array}$$

α represents the learning rate in Equations (16) and (17).

#### Theoretical Basis for CNN and DL

**Figure 2:**
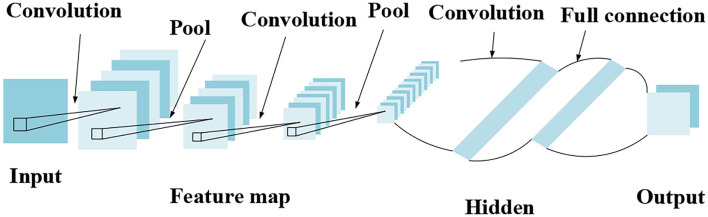
Structure of CNN.

Convolutional neural network is mainly used to extract feature information. It can effectively combine the training process and feature extraction, and has good results in image processing. Figure 2 shows the structure of CNN.

Figure 2 implies that CNN is mainly composed of an output layer, a fully connected layer, a pooling layer, a convolutional layer, and an input layer.

The convolutional layer is the core component of CNN and contains a certain number of convolution kernels. Convolution is similar to a filtering operation and is mainly used to extract features. The CNN is an inter-layer structure, which uses neurons between layers to connect. After the original image is input, it performs certain operations with the convolution kernel to output certain characteristic information. The main function of the pooling layer is to improve the generalization ability of the neural network model, while preventing the occurrence of over-fitting. The fully connected layer is usually set at the end of the network structure, and the convolutional layer and the pooling layer are connected to synthesize the extracted two-dimensional feature vectors and form a one-dimensional feature vector. Each neuron in the fully connected layer is fully connected to all neurons in the previous layer to integrate local information in the network, so it occupies most of the parameters in the network. In actual use, the fully connected operation can still be implemented by a convolution operation. The main difference between the fully connected layer and the convolutional layer is that the neurons of the convolutional layer and the input data are locally connected, and the neurons share parameters.

There are many application frameworks for DL. Among them, the Caffe framework (Somu et al., 2021) can be well combined with CNN, which is widely used in solving image problems. The Caffe framework stores data and builds training and network core units, and finally calculate the loss to get the function of a given task.

### Design of the System of Functional Strength Training Based on AI

Adopting a client/server architecture, football players' action information is collected and transmitted to convert the information into optical flow diagrams for action recognition, and finally the analysis results are obtained (Zhang et al., 2020). Camera equipment is mainly used to capture the action videos of the players to achieve the purpose of intelligent functional training for football players. The coach only needs to analyze the results in the server to get the training effect of each football player.

The design system includes two modules, the business processing module, and the video image processing module. The specific content is shown in Table 1.

**Table 1:** The specific content of the design system.

**Design system module**	**Specific content**
Service processing module	Training information storage query, identity information authentication and data processing
Video image processing module	Face recognition, motion recognition system, scoring system

Table 1 suggests that the two modules cooperate with each other to finally complete the intelligent functional training for football players.

**Figure 3:**
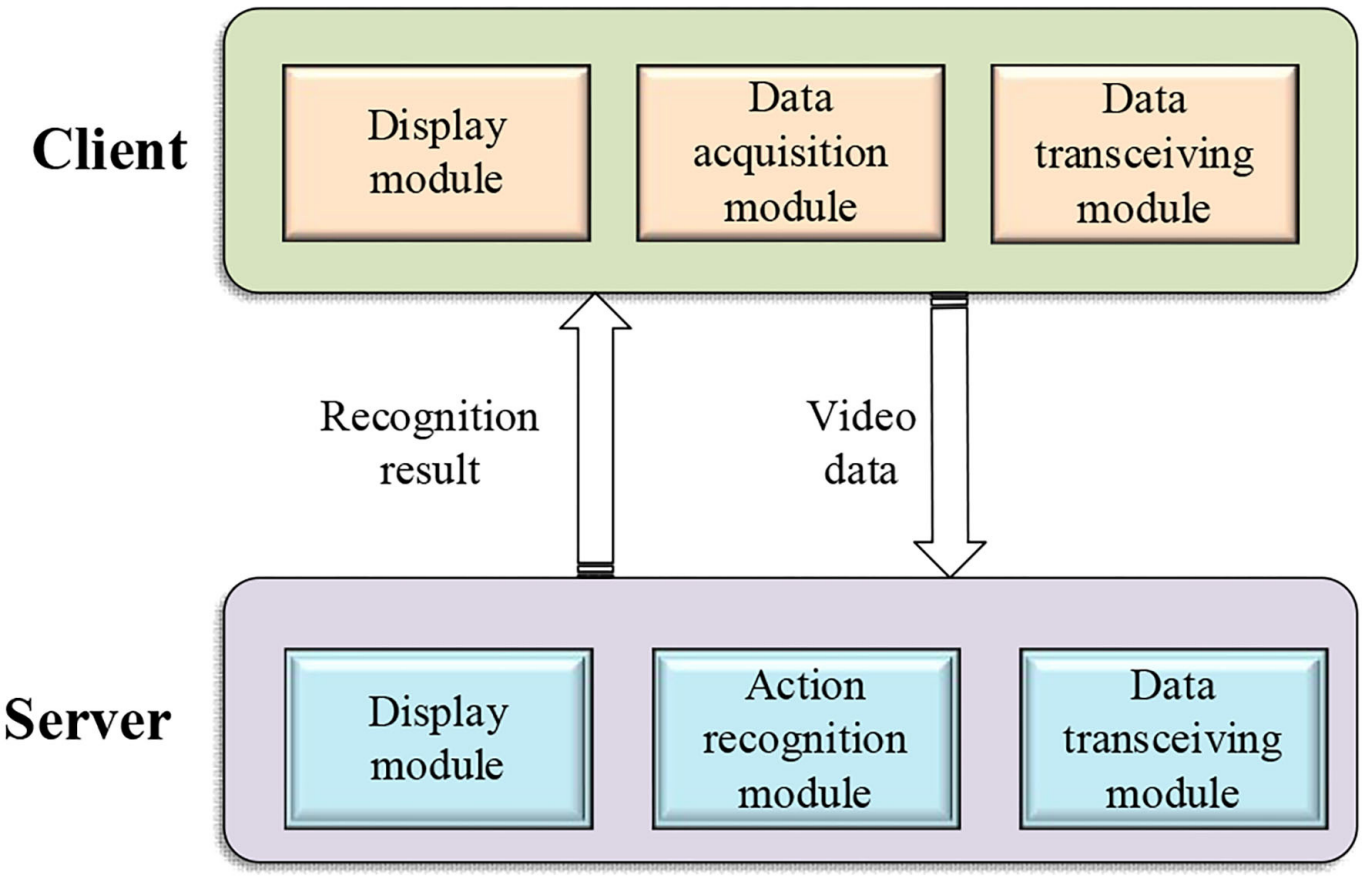
The overall framework of the design system.

The overall framework of the design system is shown in Figure 3.

Figure 3 reflects that the program of server-side recognizes and synchronizes the video data of the football players' training actions, and finally obtains the action recognition and feature extraction of the video data. The client program collects video data, then accepts the results of data recognition, and finally displays the training results of each athlete.

**Figure 4:**
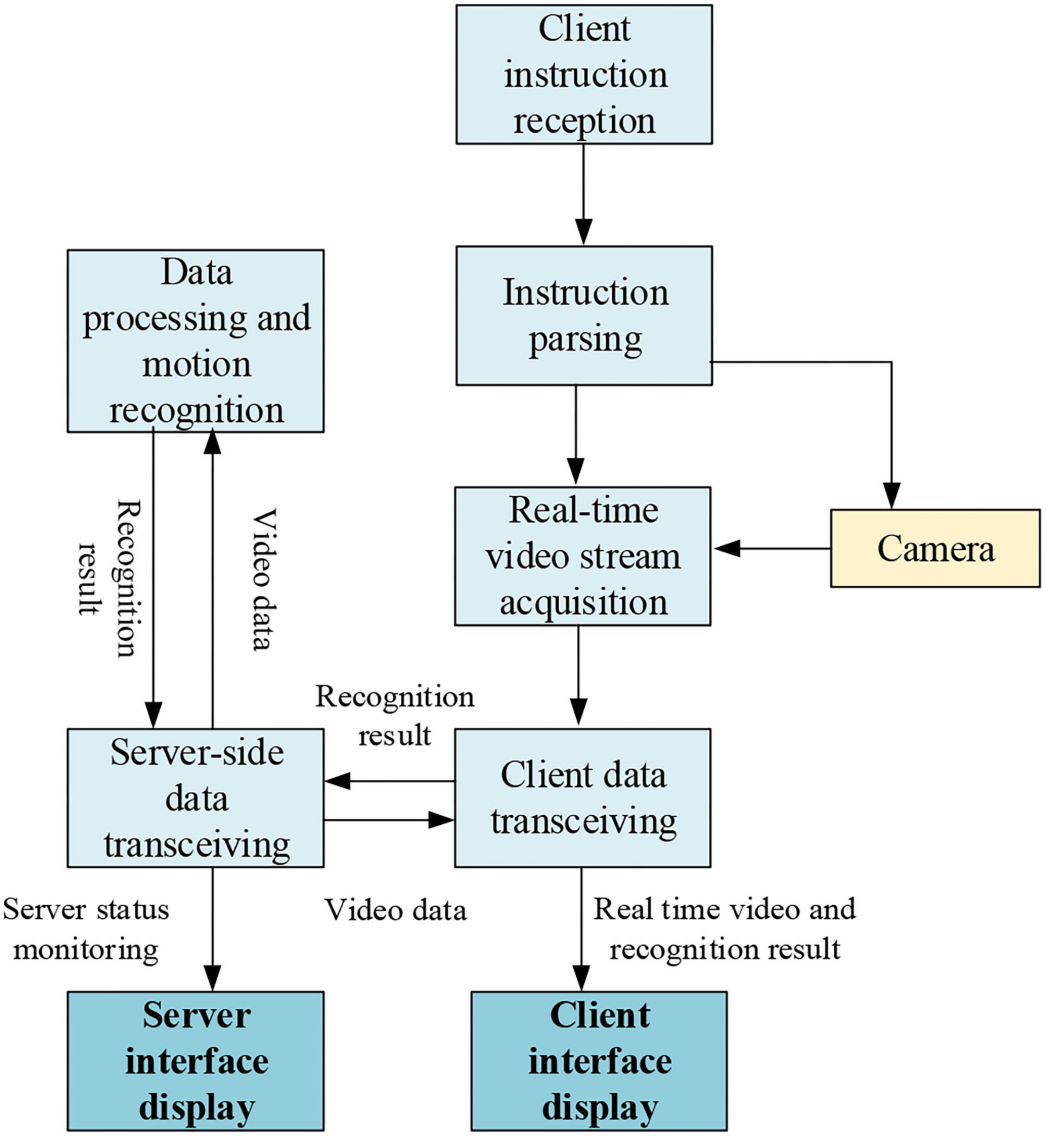
The specific workflow chart.

The specific workflow chart of the whole system is shown in Figure 4.

Figure 4 shows that the client has instructions for the training start. Once the training starts, the client performs camera shooting and synchronizes the video data to the server. The server starts the action recognition at the same time. During the training process, the client can accept user instructions throughout the entire process to stop training. The server finally feeds back the results. After receiving feedback, the client compares the recognition result of the action in the video with the training mode in the database, displays the completion of training on the client interface, and saves the data (Pareek and Thakkar, 2020).

**Figure 5:**
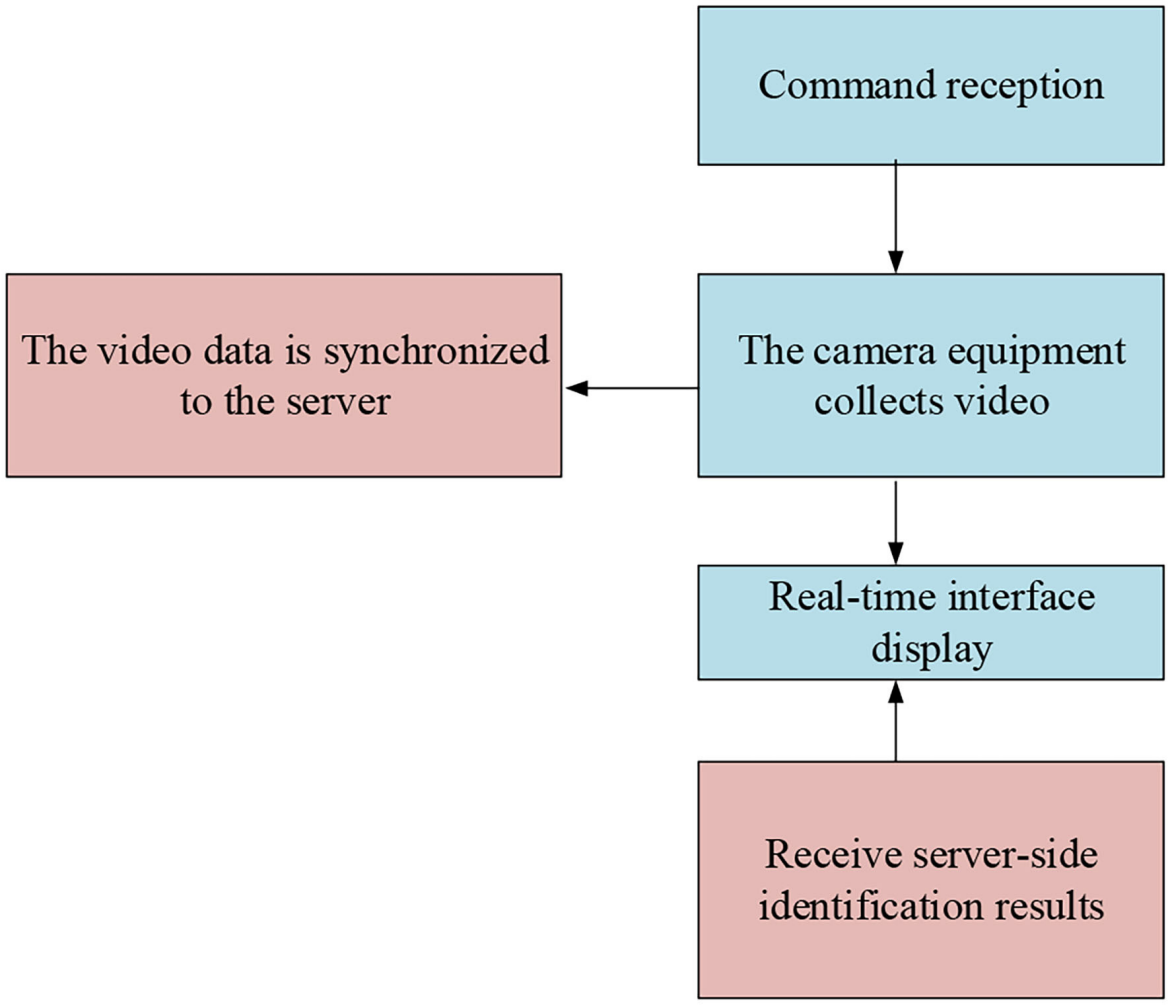
The client software workflow.

The main content of the client software workflow is shown in Figure 5.

Figure 5 presents that the client is composed of five parts. The client starts to work after receiving the start instruction. After the training video is obtained, it is synchronized to the server port immediately. After the server feeds back the recognition result, it performs real-time show on the client section interface.

**Figure 6:**
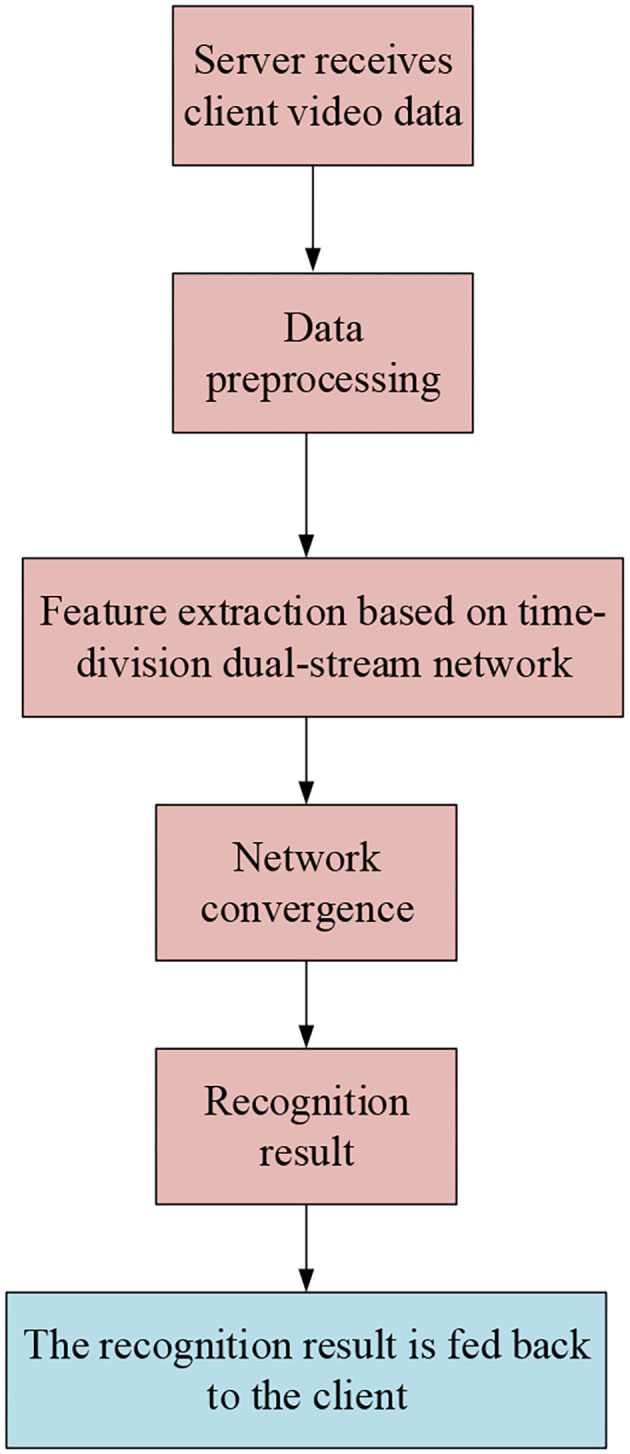
The server-side software workflow.

The server-side software workflow is shown in Figure 6.

Figure 6 shows that the server-side workflow consists of six parts. After receiving the video data, the server-side starts preprocessing the data and extracting feature, then performs network fusion, and finally feeds back the recognition results to the client terminal for interface displaying.

Since the time of data transmission of Socket is short and the amount of data is small, the communication protocol among the modules in the design system adopts Socket (Elharrouss et al., 2020).

**Figure 7:**
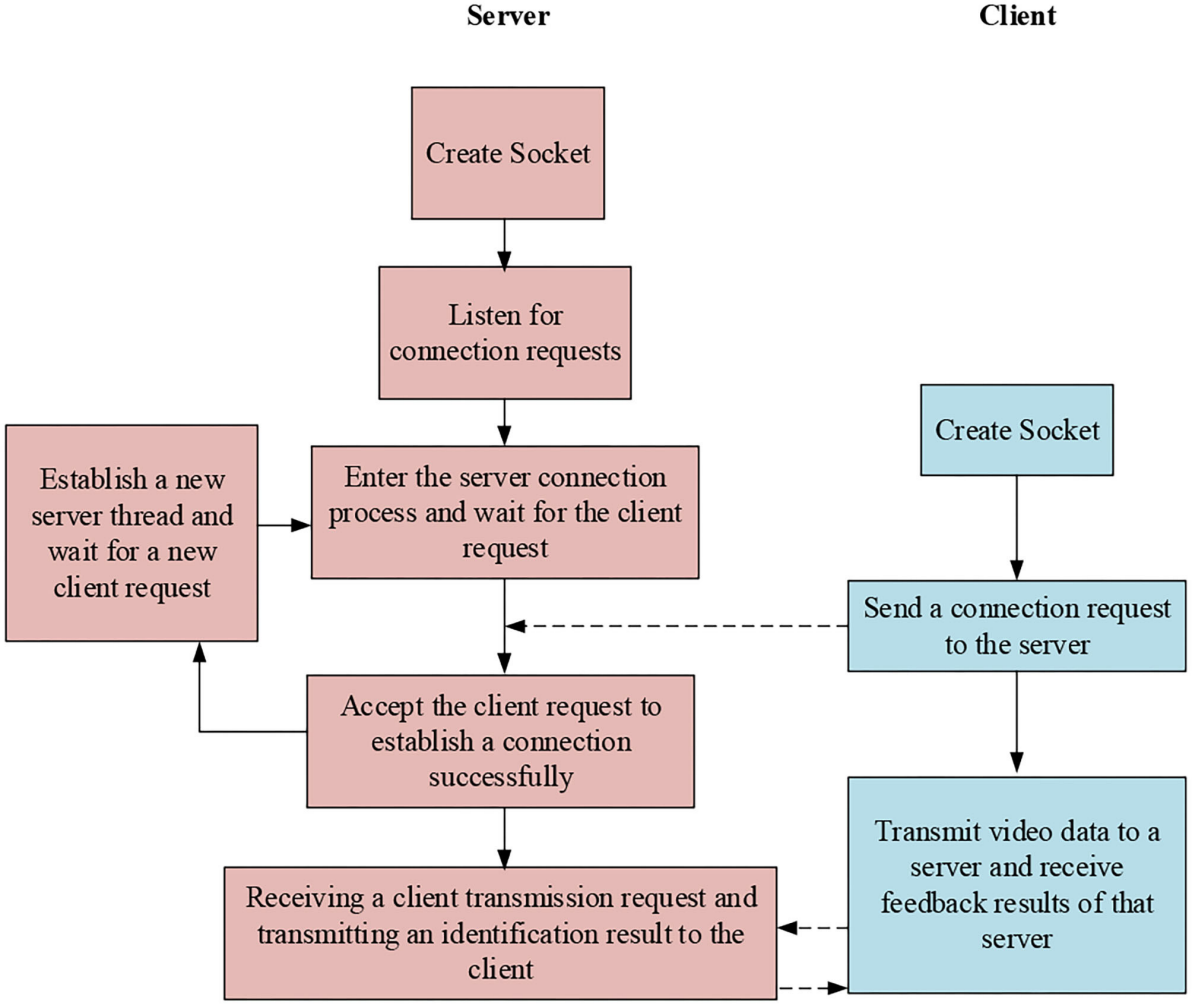
The overall communication connection of the functional strength training system.

Based on the above content, the overall communication connection of the system of functional strength training is summarized, and the results are shown in Figure 7.

Figure 7 indicates that the overall process of communication connection of the system of functional strength training strengthens the connection between the client and the server segment, which makes the video information processing faster, and at the same time, the server continues to establish new threads to ensure a complex client service demand resolution.

### Video Action Recognition

In recent years, most research has been inspired by the dual-stream CNN, which combines the spatiotemporal information extracted from the Red Green Blue (RGB) image and the optical flow image of the video, and extracts and recognizes two types of features from separate CNNs. Finally, video information generated from the prediction result contains two parts: spatial information and time information. Compared with static images, the time series components of video provide additional time-representing motion information for action recognition. The spatial information in the video is the position on each frame of the image, which represents the spatial information, such as the target and scene in the video; the temporal information refers to the change between video frames and carries the movement information of the object between video frames, including the movement of the camera or movement information of the target object, etc. The idea of recognizing video action mainly includes two categories, namely, the method of extracting video spatiotemporal features for video recognition, and the method of retraining by using human skeleton node information as network input data. The system uses the ordinary camera to collect motion video for recognition, so the dual-stream CNN is mainly used to extract the temporal and spatial features of the video for action recognition and analysis. The streaming CNN is composed of a convolutional network that expresses two-dimensional information of spatial flow and time-domain flow. It is used to process the spatiotemporal information of video data. The design architecture of dual-stream networks comes from the dual-stream hypothesis in the research of biological vision systems. Both streams are CNNs with the same structure. The spatial stream network takes a single-frame RGB image of the video as input. The decoupling of the spatial stream and the temporal stream network also enables the use of image data on large image data sets. The pretraining of the spatial stream network is used to identify the surface features related to the action and describe the feature in the spatial domain in the video. The spatial flow network is the same as the common static image recognition network, while the network of time flow inputs multi-frame stacked flow images into the network for training, and is used to learn the time features contained in the action, such as the movement and deformation of the target. It also describes the characteristics of video actions in the time domain. Using the method of multi-task training to provide two output layers of softmax for fusion, the output of the softmax layer is the probability of identifying the action category, and providing two softmax outputs is equivalent to the process of regularization. There are two main fusion methods: averaging and using the softmax layer to retrain a support vector machine (SVM) classifier for recognition.

This section gives a detailed description of the core of the research, namely video action recognition. Based on the previous content, a dual-stream CNN is used to study the recognition of remote mobilization training actions. Optical flow is the velocity vector of human motion in the video. The optical flow image is extracted from the video for data processing and motion feature extraction.

The most famous methods for calculating optical flow are the Hom—Schunck algorithm and the Lucas—Kanade algorithm. The Hom—Schunck algorithm is based on the fact that the gray level of the object image remains unchanged in a short time interval, assuming that the velocity vector field changes slowly in a given neighborhood, and then the calculation of optical flow under global smoothness constraint of the optical flow field is proposed. However, due to the smoothness assumption that the algorithm is based on, the vector estimation of optical flow may have a large error in the edge area of the image or the presence of occluded areas. The Lucas—Kanade algorithm uses local smoothness constraints, that is, assuming that all pixels in a small neighborhood have similar motions, and then realize the estimation of optical flow. Compared with the previous algorithm, the Lucas—Kanade algorithm is simple to implement, and it has lower computational complexity. After comparison, the Lucas—Kanade algorithm is selected to calculate the optical flow (Pratama, 2021). The specific calculation equation of optical flow is shown in Equation (18).


(18) $$\begin{array}{l} D^{T}D\overset{\to }{w}=D^{T}\left(-c\right) \end{array}$$

In Equation (18), *T* refers to time; *c* is a constant term; and *D* is a coefficient matrix, *w*=*dy/dt*, where y is the ordinate position of the action. Solving Equations (18) and (19) (Zhao et al., 2021) can be obtained.


(19) $$\begin{array}{l} \left[\begin{array}{c} u\\ w \end{array}\right]={\left[\begin{array}{c} \sum_{i}F_{x}{\left(X_{i}\right)}^{2}\&\sum_{i}F_{x}\left(X_{i}\right)F_{y}\left(X_{i}\right)\\ \sum_{i}F_{x}\left(X_{i}\right)F_{y}\left(X_{i}\right)\&\sum_{i}F_{x}{\left(X_{i}\right)}^{2} \end{array}\right]}^{-1}\left[\begin{array}{c} -\sum_{i}F_{x}\left(X_{i}\right)F\left(X_{i}\right)\\ -\sum_{i}F_{y}\left(X_{i}\right)F_{t}\left(X_{i}\right) \end{array}\right] \end{array}$$

In Equation (19), *u*=*dx/dt, F* is the brightness, and *x* is the abscissa of the position of the action.

Data collection uses Haikang camera equipment to obtain the video, and then uses the kit for software development of equipment network to convert the information flow into a three-color mode, and finally uses a sparse sampling strategy to process the video information. The collection process of real-time video stream first needs to initialize the software development kit (SDK). The device network SDK is developed by Haikang based on the communication protocol of private networks. It is a supporting module for network hard disk recorders, video servers, network cameras, and other products. It can be used for remote visit equipment and conduct secondary development. It could verify and login by setting the Internet Protocol (IP), port number, and other information of the webcam; then real-time video preview could be realized by setting the playback channel, video stream types, connection methods, and other information. It could be realized that the real-time video stream acquisition is done through the playback callback function; in addition, setting the connection timeout, reconnection time, and abnormal state callback functions need to be done; finally, the SDK is called to convert the obtained real-time video stream into Red, Green, Blue, Alpha (RGBA) data, and the entire video stream collection is completed. After using the Haikang camera to obtain the real-time video stream, the data transmission module on the client end will synchronize the video stream data frame by frame to the server-side program for processing, and save it as a video file in the client hard disk at the same time.

Based on the previous content, the image data set the size of the input training of the network design as 224 × 224. Through the first layer of convolution conv1, the size of the convolution kernel is set to 7 × 7, and the step size is set to 2, with a total of 96 convolution kernels, outputting 96 feature maps of 112 × 112 size. After the pooling operation, 96 feature maps with a size of 56 × 56 are output. Then through the second layer of convolution, conv2, the size of the convolution kernel is set to 5 × 5, and the step size is set to 2, and a total of 25 convolution kernels and 256 feature maps with a size of 28 × 28 are output. After the pooling operation, 256 14 × 14 feature maps are output. Then through the third layer of convolution, conv3, the fourth layer of convolution, conv4, and the fifth layer of conv5 are output. If the size of the convolution kernels of the three layers are all 3 × 3, the step size is also 1, and the number is also 512, so that after the last three layers of convolution, 512 14 × 14 feature maps are output. Then, after the pooling operation, 512 7 × 7 feature maps are output. Finally, through the two fully connected layers, full 6, full 7 (global average pooling avg pool), and the final Softmax, the output is obtained as a 1 × 1 × 101 or 1 × 1 × 51 vector. The main content of the functional training database for football players involved in this study is shown in Table 2 (Cournoyer et al., 2021).

**Table 2:** Actions of functional training database.

**Functional strength training method**	**Specific requirements**
Jumping power	In groups of three, tie sandbags on your legs to fight for the top ball
Kicking strength	After finishing the forward roll, jumping the low hurdle and drilling the high hurdle, sprint and shoot
Power in competition	Two football players cooperated, one dribbled the ball quickly, and the other made a tackle. After the tackle was completed, he quickly got up and chased the ball
Starting force in running	In groups of 10 people, after hearing the whistle, they began to sprint 100 meters
Leg strength	In groups of two, one person throws a solid ball to the other party, and the other party passes it back with his feet after receiving the ball

Table 2 presents that the functional strength training of football players is mainly carried out from the aspects of jumping strength, kicking strength, and competing strength. The training video in the database of this design system is obtained by referring to the current football player training video. After obtaining the real-time video, the computer is used to compare the training actions in the video with the actions in the database to obtain the quantified scores of speed and strength.

**Figure 8:**
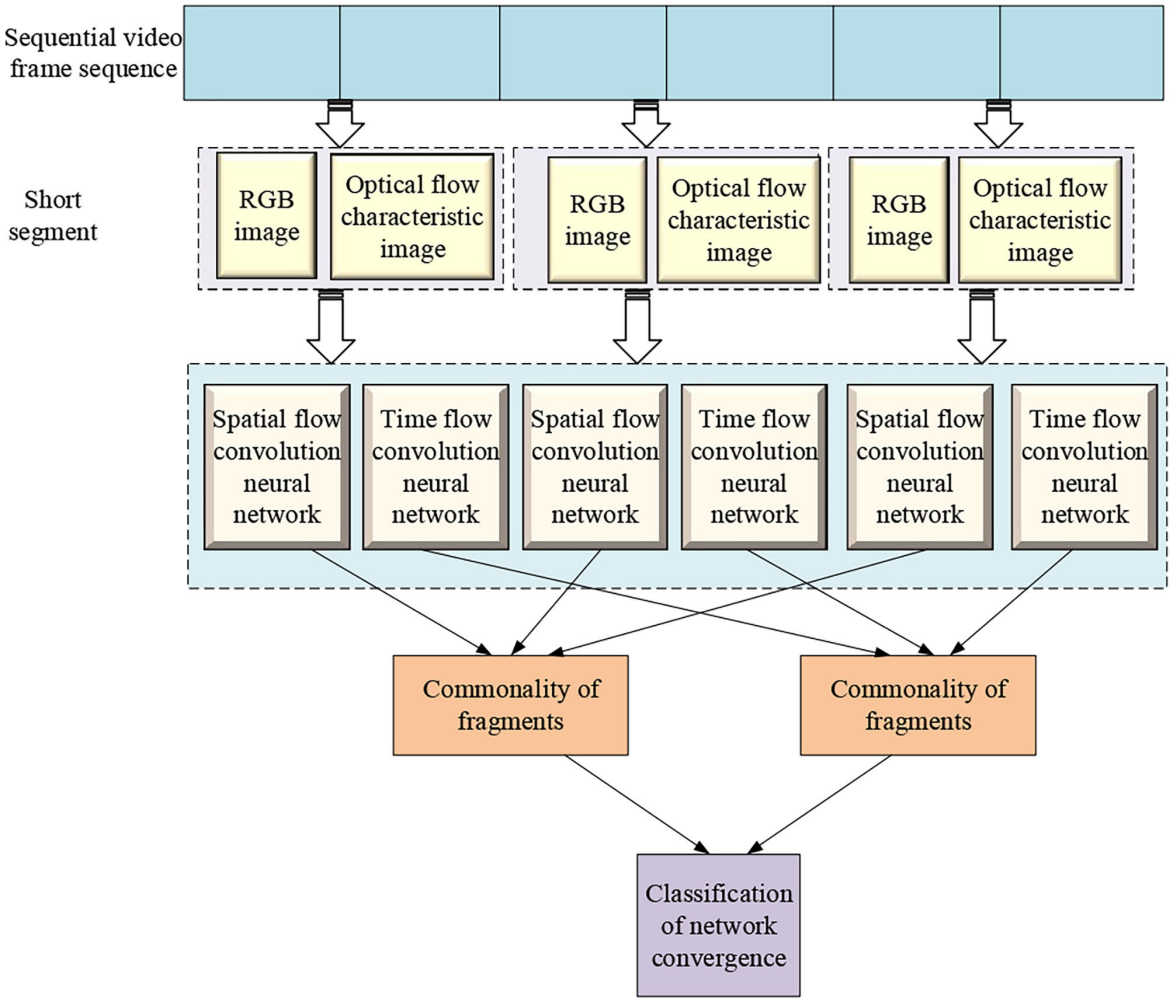
The overall architecture of the network model of action recognition.

Based on the above content, the overall architecture of the network model of action recognition is given here, as shown in Figure 8.

As Figure 8 suggests, a time-segmented CNN is used based on a sparse sampling strategy. The time-segmented CNN is used to segment the entire video and sparsely sample short segments from it as the network input. The temporal characteristics of optical flow images and the spatial characteristics of RGB images are extracted to perform action recognition. The time segmentation CNN first divides a video containing an action into several equal parts, and then randomly extracts a short sequence from it, so that the short segment generated by its sampling can effectively express the motion information in the entire video. For each sampled segment, feature extraction is performed through a dual-stream CNN. The temporal stream network captures the temporal structure information of the video and the spatial appearance information of the image captured by the spatial stream network, and it generates a corresponding dual-stream network prediction for each short segment. Finally, an aggregation function is used to fuse the characteristics of time flow and spatial flow network as the recognition result of the whole video. This method can effectively extract the long-term information of the entire video, which is more accurate and effective than the recognition method by densely sampling the entire video segment and does not increase the calculation cost. In the learning process, the loss value of the entire video prediction is optimized through the iterative update parameter calculation to realize the end-to-end network training process.

The application of the research system to the speed and strength of football players will also be reflected by a scoring system. The scoring system is based on video recognition. After comparing and analyzing the standard data in the video recognition system, the scoring system will score the athletes' movements according to the differences in their movements. Since the improvement of the speed and strength of football players will be reflected in the performance during the game, the scoring system mainly deducts in accordance with the foul action of the players. The system will give the detailed foul or unqualified actions of the football player for reference and promotion.

Under the premise of using the action recognition system to identify the result of foul action, Harris3D operator is used to establishing the potential function of the foul action sequence, which provides support for foul action feature extraction. By Harris3D operator, the foul action is mined; the spatiotemporal interest points of each different foul action are extracted in the image; the features of gradient histogram and streamer direction histogram are obtained, which are divided into 72-dimensional and 90-dimensional images, respectively. Fusion gradient histogram and the streamer direction obtain a 162-dimensional feature vector based on the histogram, which constitutes the underlying feature of the foul action and selects the key skeleton points of the football player. According to the characteristics of human body structure, the football player's body is divided into seven local reference points: shoulders, left and right arms, left and right legs, and left and right feet. The shortest Euclidean distance between each point of interest in time and space of the foul action and the local reference center point is shown in Equation (20).


(20) $$\begin{array}{l} n=\frac{\mathrm{argmin}\sqrt{{\left(a_{i}-x_{j}\right)}^{2}+{\left(b_{i}-y_{i}\right)}^{2}}}{\left(x_{ṅ},y_{n}\right)} \end{array}$$

In Equation (20), *n* represents the local reference center human body range mark, which is called the spatiotemporal interest point range. It is divided into 7 regions according to the local reference point; *(xj, yj)* represents each spatiotemporal interest point.

The foul action set time and space interest points are divided into three levels, which are the characteristics of all foul actions of football players; the corresponding foul action features of the shoulder, left and right arms, and left and right legs; the foul action characteristics of the shoulder, left and right arms, left and right legs, and left and right feet. Using *K*-means to cluster the bottom foul action features, the number of cluster centers is *K*, and *K*×162-dimensional foul action images are obtained. At the same time, three levels of foul action images in seven areas are generated, and the T frame of the foul action in each area is defined as a foul action spatiotemporal action module, which represents a certain part of the foul action feature of a football player. The number of overlapping frames of two foul action image units is *T*/2, and all action units in each foul action image are organically synthesized to obtain a spatiotemporal foul action unit sequence with a length. When comparing the sequence data extracted from standard actions and actions of football players, since everyone's action sequence time is not necessarily the same, the direct use of conventional distance comparison methods, such as Euclidean distance will result in poor scoring accuracy, so this method of sequence comparison is adopted to match the dynamic time warping (DTW) algorithm.

### Simulation Experiment of Intelligent System of Functional Strength Training

It can be seen from Figure 8 that after the video segment is input to the algorithm, the tricolor mode image and the optical flow image are first extracted (Fan et al., 2020), and then combined with the commonality of the segment to classify the video action, and finally the video recognition result is obtained.

The feature fusion method is convolution fusion, and the fusion is achieved through the combination of two CNNs after feature extraction. The dual-stream fusion adopts pooling and three-dimensional convolution.

Shen et al. (2019) proposed a method for mining the text of two-layer concept link analysis. Based on this, a data set is selected. The data set contains 4,800 videos with a resolution of 320 × 240, and the duration of each video action is 3–9 s, 25 frames per second. The content of the videos are presented in Table 2. First, the grid training of the gradient descent algorithm is performed, the parameters are set to a momentum of 1.0, and the batch size is 256 (Wang et al., 2019). The parameters of the dual-stream network set as the initial weight of time flow is 0.01, and the space flow is 1. Three methods are used for experimental verification, namely, time flow, and space flow network zeroing training, the CNN of only pretraining the space flow, and initializing the time flow network using the tricolor mode to compare the recognition effect of the dual-stream CNN. On this basis, the commonly used quantity set is introduced, and the recognition effect between the algorithm designed herein and other common algorithms under the two kinds of quantity sets is compared.

Finally, the practicability and reliability of the entire system are verified. About 20 football players are selected to collect and verify 5 training actions. Finally, the best result of each recognized action is selected. The recognition accuracy greater than 90% is considered an excellent one. If the recognition accuracy is between 85 and 90%, it is good (Gong et al., 2019).

Due to the addition of a scoring system in the experiment, the performance of football players will be reflected in scores. The scores under the traditional training mode are counted by the manual score of the football team coach, and the training system with AI is scored by the computer system. The training improvement is reflected by the football action score improvement rate before and after the use of the two training modes.

The operating system of the experimental environment adopts the operating system (CentOS) Linux release 7.3.1611, the central processing unit (CPU) adopts Intel Xeon E5-2680 v2. It has the memory capacity of 128 Gigabytes (GB), double data rate (DDR) of 2 2,400/3,200MHz, and hard disk capacity of 512 GB+4 Terabyte (TB). The graphics processing unit (GPU) used is GeForce Giga-Texel (GTX) Titan V.

### The Algorithm Design Effect Is Compared With Traditional Training

Since the purpose is to compare with traditional strength training to improve the speed and strength of football players, a comparative experiment is carried out here. About 20 athletes are selected and divided into two groups. They are given a traditional functional strength training for a week, and based on that, an algorithm has been designed herein. Finally, the speed and strength results of the two groups before and after training are tested. The intelligently trained athletes are scored by statistics, and the ability of athletes under traditional training are scored by coaches. Five kinds of action are set up to improve the speed and strength of athletes.

The learning of network parameters is realized by a small batch of gradient descent algorithms, with batch size set to 256 and momentum set to 0.9. Gradient descent in small batches can be accelerated by the calculation of matrices and vectors, and the variance of updated parameters are reduced to obtain more stable convergence. Using a batch each time can reduce the number of iterations of convergence, and at the same time make the result of convergence closer to the effect of gradient descent. For the traditional descendent gradient algorithm, if the functional plane of the actual objective is a partially concave surface, then a negative gradient will make it point to a steeper position. This situation near the local optimal value of the objective function will slow down the convergence speed. At this time, it is necessary to give the gradient a momentum, so that it can jump out of the local optimum and continue to optimize in the direction of the gradient descent, so that the network model can more easily converge to the global optimum. For the time segmentation dual-stream network used in this system, the initial weight of the spatial stream convolutional neural network is set to 1, and the initial weight of the time stream convolutional network is set to 1.5. The learning rate for the network training is set smaller: the initial value of the spatial flow of CNN is set to 0.01, and is adjusted to one tenth for every 2,000 iterations; the initial value of the time flow of CNN is set to 0.005, and is adjusted to one-tenth after 12,000 and 18,000 iterations of network parameters. In addition, the total time-consuming data training are as follows: the spatial streaming network requires about 2 h; the time streaming network requires about 11 h.

There may be a risk of overfitting when deep CNN is used for network training. In order to further alleviate this problem, three network training strategies are used here to compare the ability to alleviate the risk of overfitting. The first method is to train the spatial stream and time stream network directly from scratch by using the Gaussian distribution to initialize the CNN parameters, without any other means of pretraining processing; the second method is to perform the pretraining processing for convolving the spatial stream CNN. Since the spatial stream convolutional network only uses RGB images as the network input data, the convolutional network can be pretrained through the Image Net image database, and the pre-rained network parameters are used as the initial parameters of spatial stream network; the third method is the pretraining processing method of the cross-input model that initializes the time flow network with the RGB model, while still initializing the Image Net data set as the pretraining input data of the spatial flow network. First, the image pixels are deep CNN discretized at the interval of [0, 255] through linear changes of optical flow characteristics, so that the range of the optical flow image is the same as the RGB; then the mapped image is input to the spatial flow convolutional network for training, and then the preprocessing is performed. The weights of the first convolutional layer of the trained spatial stream convolutional network model are averaged, and the average value is copied according to the number of input channels of the time stream convolutional network. The time-splitting dual-stream network divides the input video sequence into three segments for processing. Therefore, the average value is used as the initial weight of the three channels of the time flow network for training.

### Field Line Detection for Football Robots

**Figure 9:**
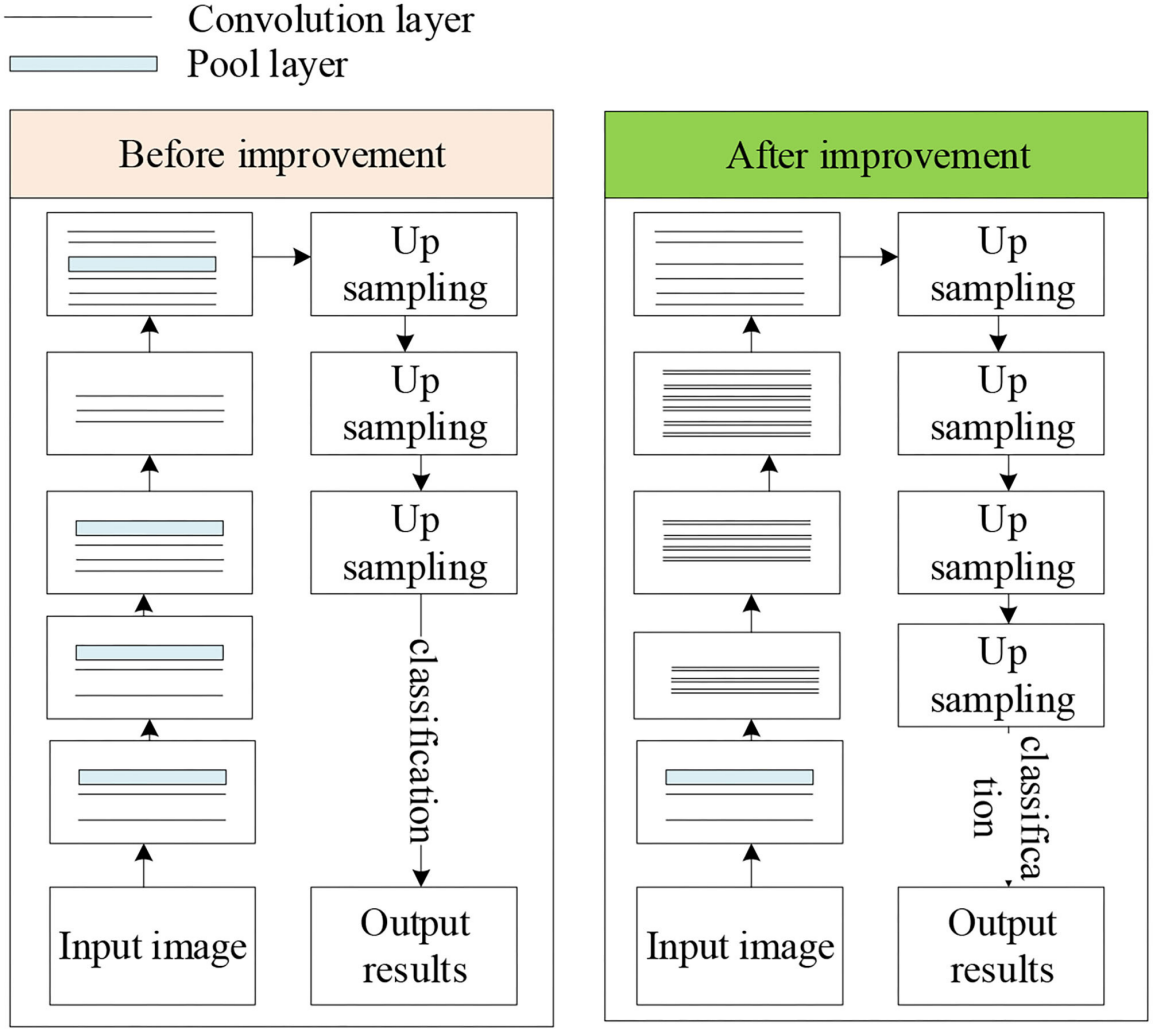
The model before and after improvement.

Based on the previous content, combined with CNN for yield line detection, but the extraction ability of traditional FCN model is weak, so it needs to be improved. By increasing the number of convolutional layers, introducing residual structure, constructing residual blocks, and enhancing the model feature extraction ability, the accuracy of field line detection is improved finally. After the residual structure is introduced, the input feature of each convolutional layer is the sum of the input and output features of the previous convolutional layer, so each convolutional layer can extract more features, which is more conducive to improving the detection accuracy. Residual block refers to a residual structure containing multiple consecutive convolutional layers, that is, a direct correlation channel is introduced between the input of the first convolutional layer and the output of the last convolutional layer, so that the input features of the residual block are directly added to the output features as input features for the next convolutional layer or residual block. The model before and after improvement is shown in Figure 9.

In Figure 9, compared with the FCN model, the convolutional layers of the improved model are increased from 15 to 34, and the convolutional layers are constructed into 4 residual blocks, which improves the ability of feature extraction. In the residual block, a convolutional layer with a stride of 2 is used to replace the pooling layer for downsampling. In the process of upsampling, a cross-layer connection structure is used to fuse the output feature maps of all residual blocks to make the final detection result more refined, and the field line detection can be performed more accurately.

The evaluation index of the field line adopts the average pixel precision *P* and the mean intersection over Union (MIoU) *Q*, and the specific calculation is shown in Equations (21) and (22).


(21) $$\begin{array}{l} P=\frac{1}{k+1}\overset{k}{\underset{i=0}{\sum }}\frac{\sum_{i=0}^{k}P_{ii}}{\sum_{j=0}^{k}P_{ij}} \end{array}$$

(22) $$\begin{array}{l} Q=\frac{1}{k+1}\overset{k}{\underset{i=0}{\sum }}\frac{P_{ii}}{\sum_{j=0}^{k}P_{ij}+\sum_{j=0}^{k}P_{ji}-P_{ii}} \end{array}$$

In Equations (21) and (22), *k*+*1* is the number of categories (including 1 background class); *P*_*ii*_ indicates the point that is correctly predicted; *P*_*ij*_ means that the pixel marked as class *i* is predicted to be the pixel of class *j; P*_*ji*_ denotes that the pixel marked as class *j* is predicted to be pixels of class *i*.

In the experimental field, three lighting conditions are set, namely, lighting, sufficient natural light, and insufficient natural light. The average pixel precision and MIoU of field line detection between the color segmentation model and the improved model are compared under different lighting environments, and the average pixel precision and MIoU of the FCN model and the improved model are compared. Finally, the continuously running time of three models is compared, and the model uses 100 images of the research area.

## Analysis of Intelligent Detection Results of Functional Training Intelligent System and Football Robot

### Analysis of Functional Training Results

**Figure 10:**
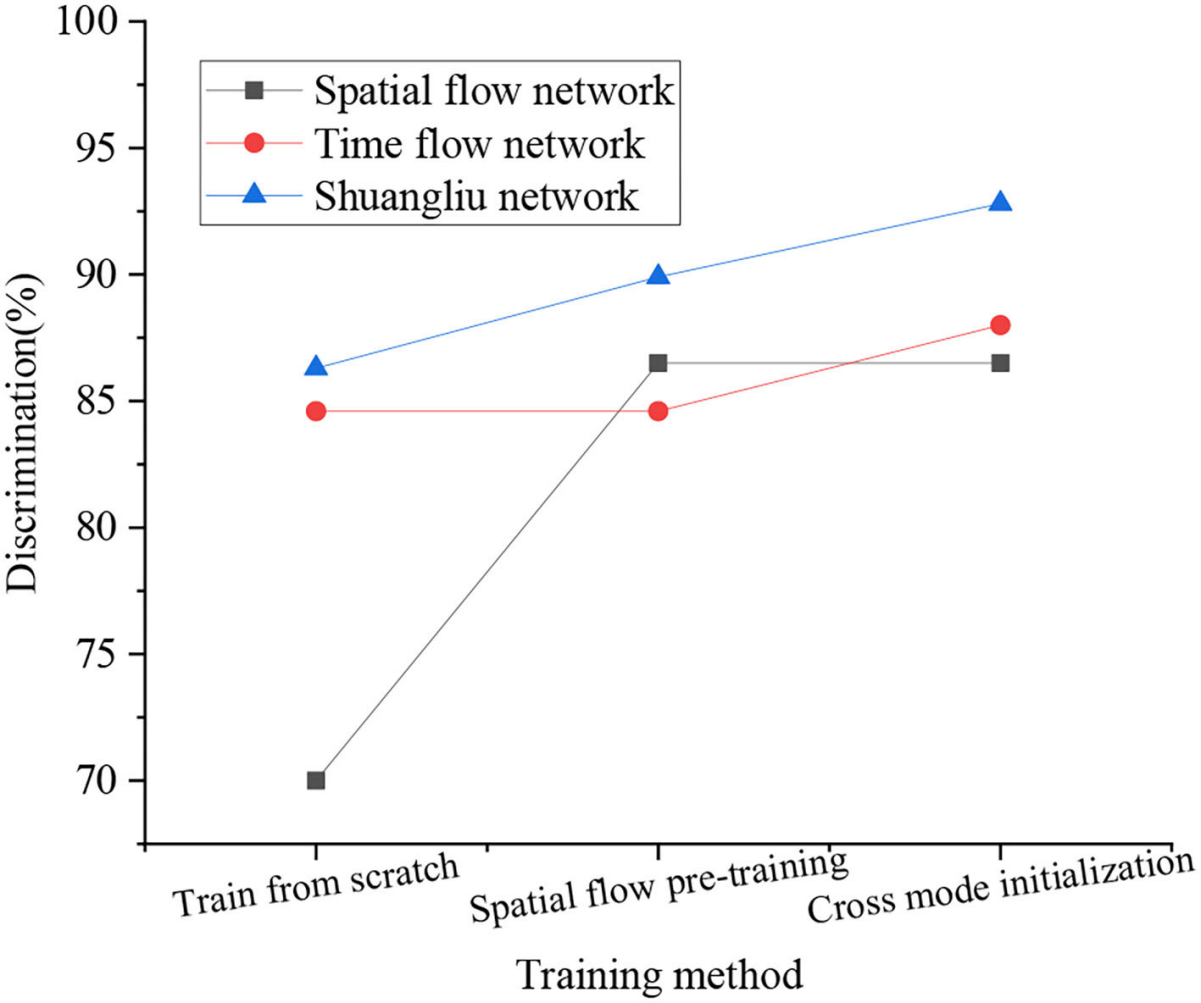
Comparison of recognition effects of CNN under different training methods.

Based on the previous content, a comparison of the recognition effect of CNN under different network training methods is obtained, as shown in Figure 10.

Figure 10 displays that under different training methods, the recognition effect of CNN is somewhat diverse. Among them, the dual-stream network has the best effect, with a recognition rate of 92.8%. On the whole, the recognition rate of the time-stream network is lower than that of the Shuangliu network, and its highest rate is 88%; the recognition rate of the spatial stream network is the lowest among the three comparisons, which is 86.5%. The overall recognition rate of the network under the three training methods is sorted from large to small, cross mode initialization, spatial flow pretraining, and training from the scratch. Analysis of the reason for this phenomenon is that the feature recognition dimension of the dual-stream network is higher, and the coverage is more comprehensive, and the single network recognition often has only one dimension. The method of training from scratch is prone to overfitting when the data set is small, so the recognition rate is the lowest among the three training methods, and the spatial flow pretraining method can reduce the risk of overfitting to a certain extent, and the training method of crossover mode could reduce risks to the largest extent. It can be seen that the design algorithm is more feasible.

**Figure 11:**
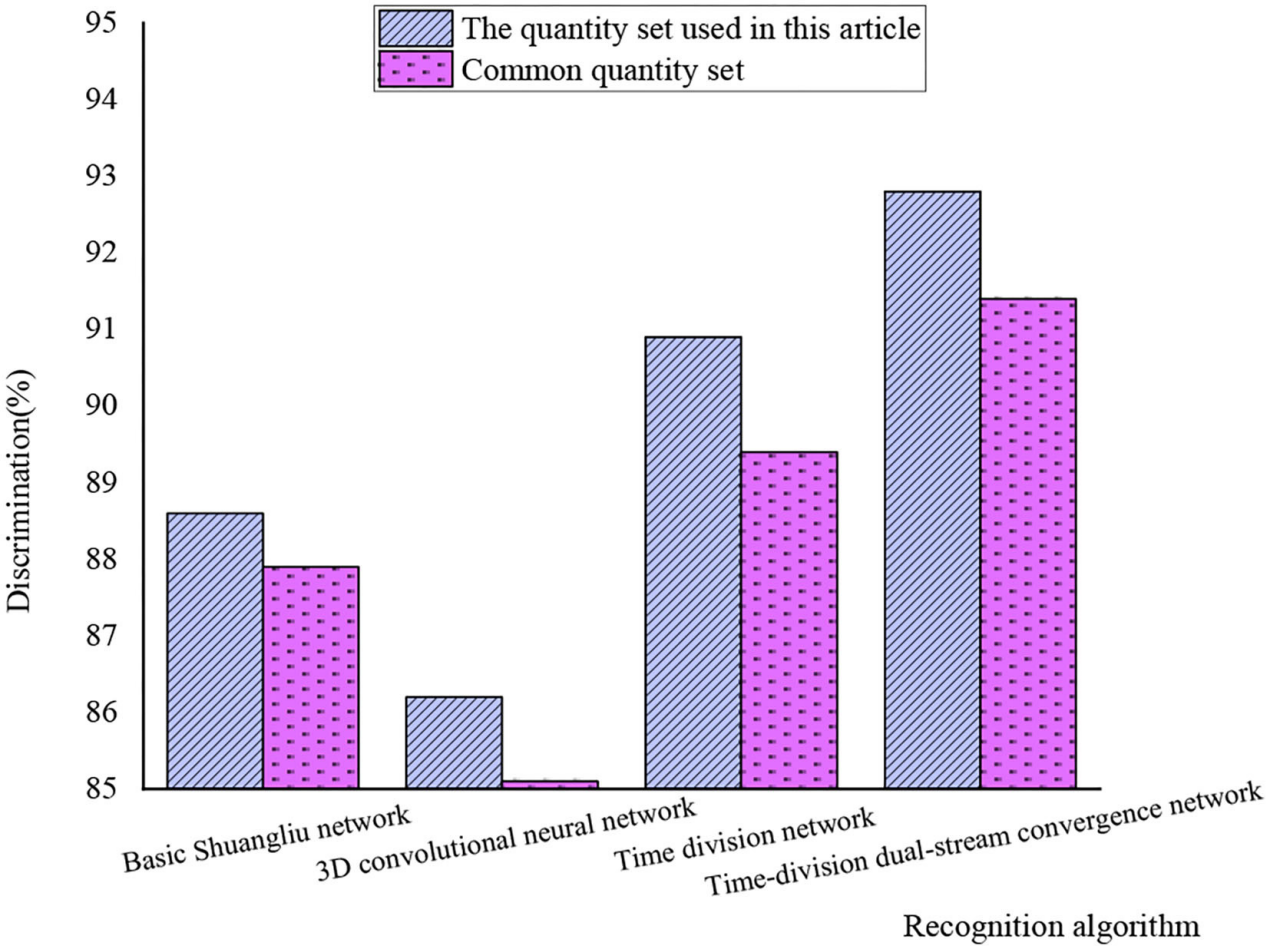
Comparison of recognition effects among different algorithms.

After the introduction of common quantity sets, the processing results of common algorithms for the two kinds of quantity sets are shown in Figure 11.

Figure 11 shows that in the processing of the data set, the four algorithms have different effects on the video set selected from herein and the common video set. The processing power of the data set herein is stronger than that of the common video set. The recognition rate of the time-divided dual-stream fusion network is the highest, reaching 92.8%. The recognition rate of the time segmentation network is second only to the network designed herein, which is 90.9%. The recognition rate of the basic dual-stream network is 88.6%, and the recognition rate of the 3D convolutional neural network is the lowest, just 86.2%. When analyzing the reasons, it can be seen that the commonly used data set has a large amount of data, so the processing effect is poor. The time-divided dual-stream fusion network can sample efficiently, and other algorithms are prone to excessive information due to a long time for sampling. The algorithm designed herein is highly reliable.

**Figure 12:**
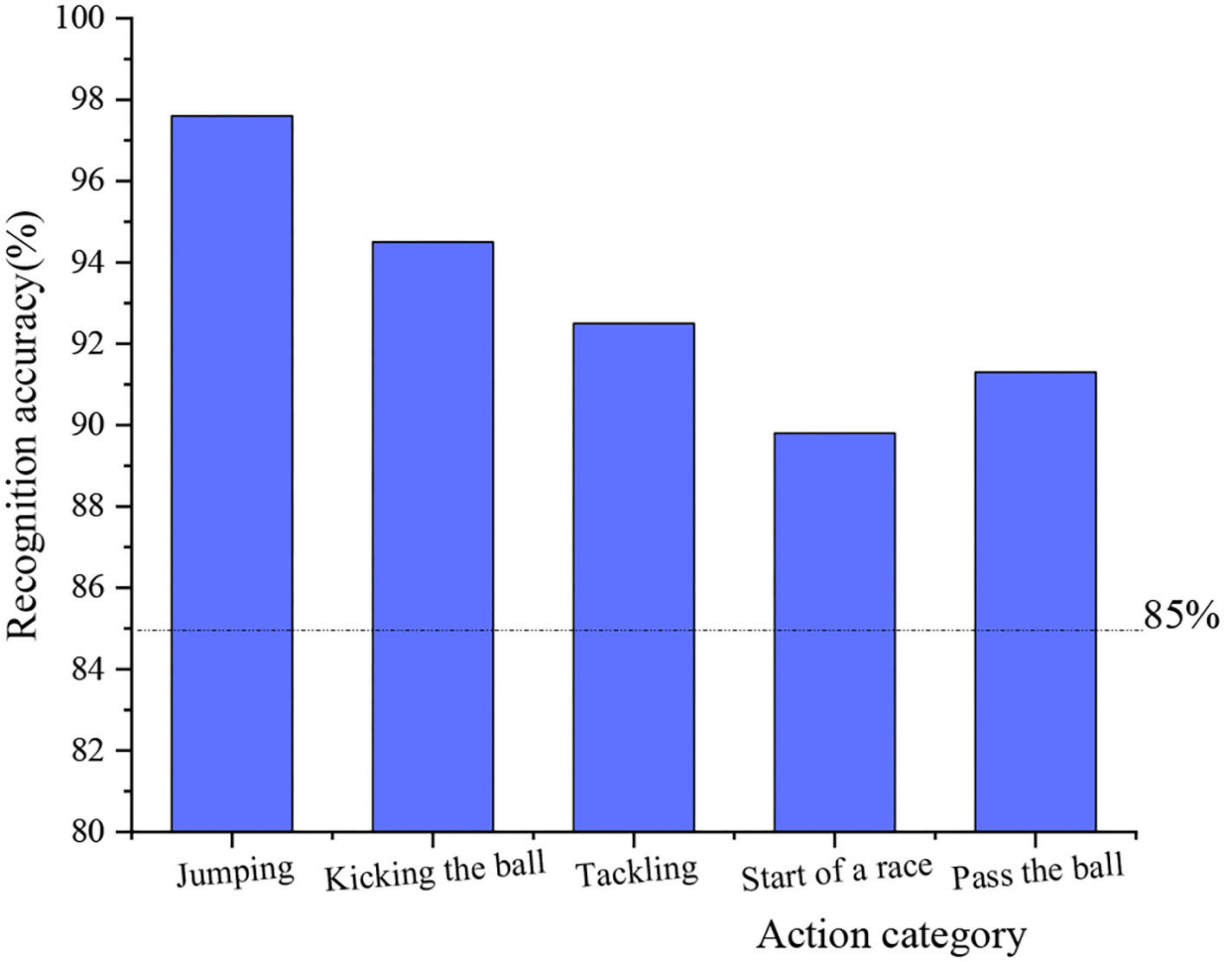
The recognition accuracy of different actions.

On the basis of Table 2, the recognition accuracy of the five actions of this design system is compared, and the results are shown in Figure 12.

Figure 12 demonstrates that under the intelligent training system, the recognition accuracy of different actions is high, and the highest is that of jumping actions, reaching 97.6%, and the recognition accuracy of kicking actions is 94.5%. Grabbing action is 92.5%, starting action is slightly lower, reaching 89.8%, and that of passing action is 91.3%. It can be seen that the recognition accuracy is beyond the good level. Except for the recognition accuracy of the starting movement, others are all at an excellent level. The analysis shows that when the 10-person group set up starts, the overlap rate of the athletes in the video is relatively high, so the system identification is more difficult. The intelligent training system can meet the design requirements.

**Figure 13:**
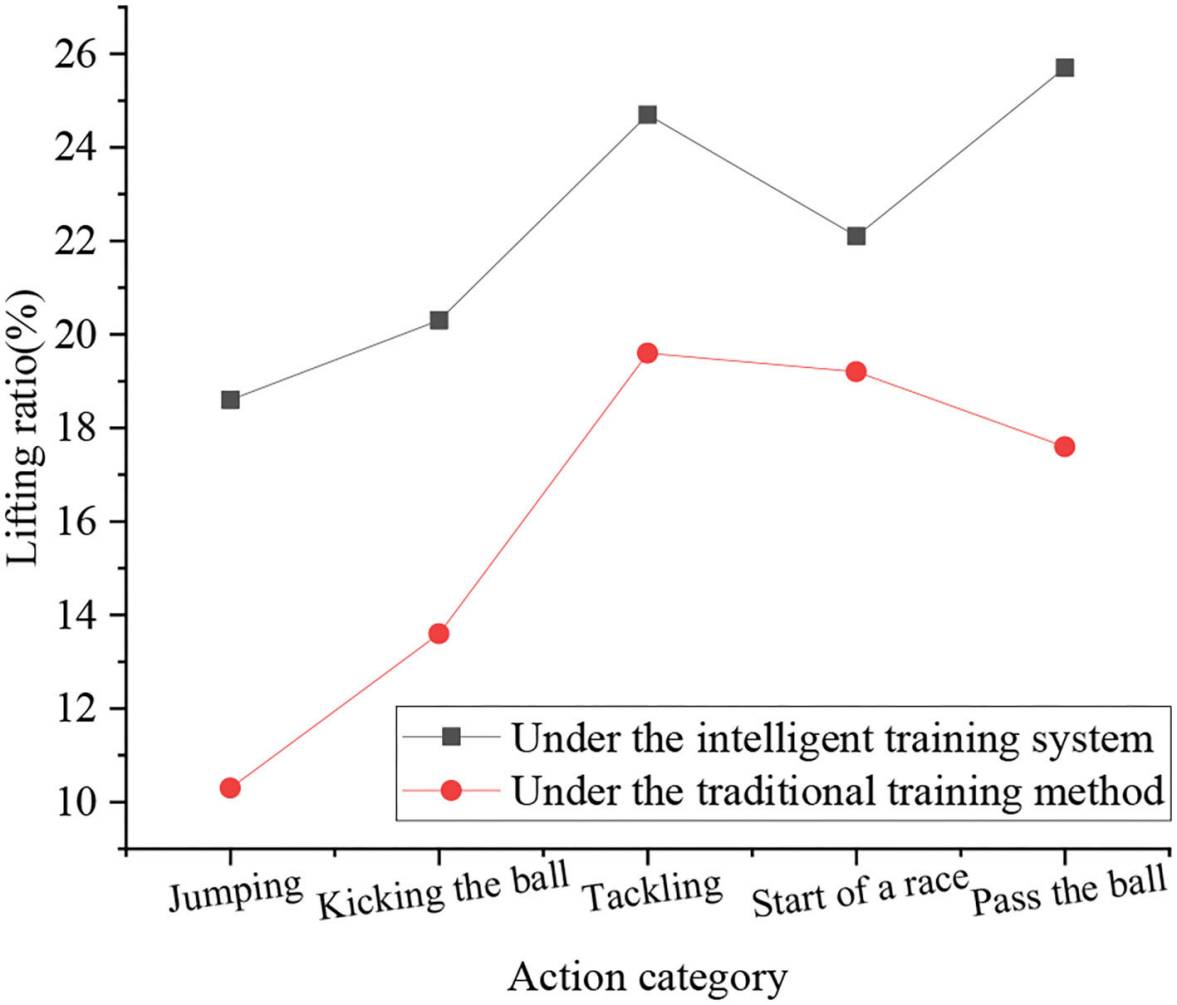
Comparison of training results.

Finally, the results of the functional strength training of the athletes under the AI system are counted, and it is compared with the scores under the traditional training methods. The results are shown in Figure 13.

Figure 13 shows that the upgrade rate of speed and strength under intelligent training is higher than that under traditional training. The maximum upgrade rate of intelligent training is 25.7%, which is the improvement of passing ability, and the minimum is 18.6%. For the improvement of jumping ability, the highest upgrade rate of traditional training is the ability to compete for the ball, which is 19.6%, and the lowest is the improvement of jumping ability, which is 10.3%. Since intelligent training strengthens the autonomy of football players, the upgrade rate increases rapidly. The experiment is set for 1 week, but it takes a long time for the training of jumping ability to be effective. Therefore, the upgrade rate of jumping ability in the two training groups is low. In summary, the intelligent training system has a strong applicability.

### Analysis of Field Detection Results of Football Robots

**Figure 14:**
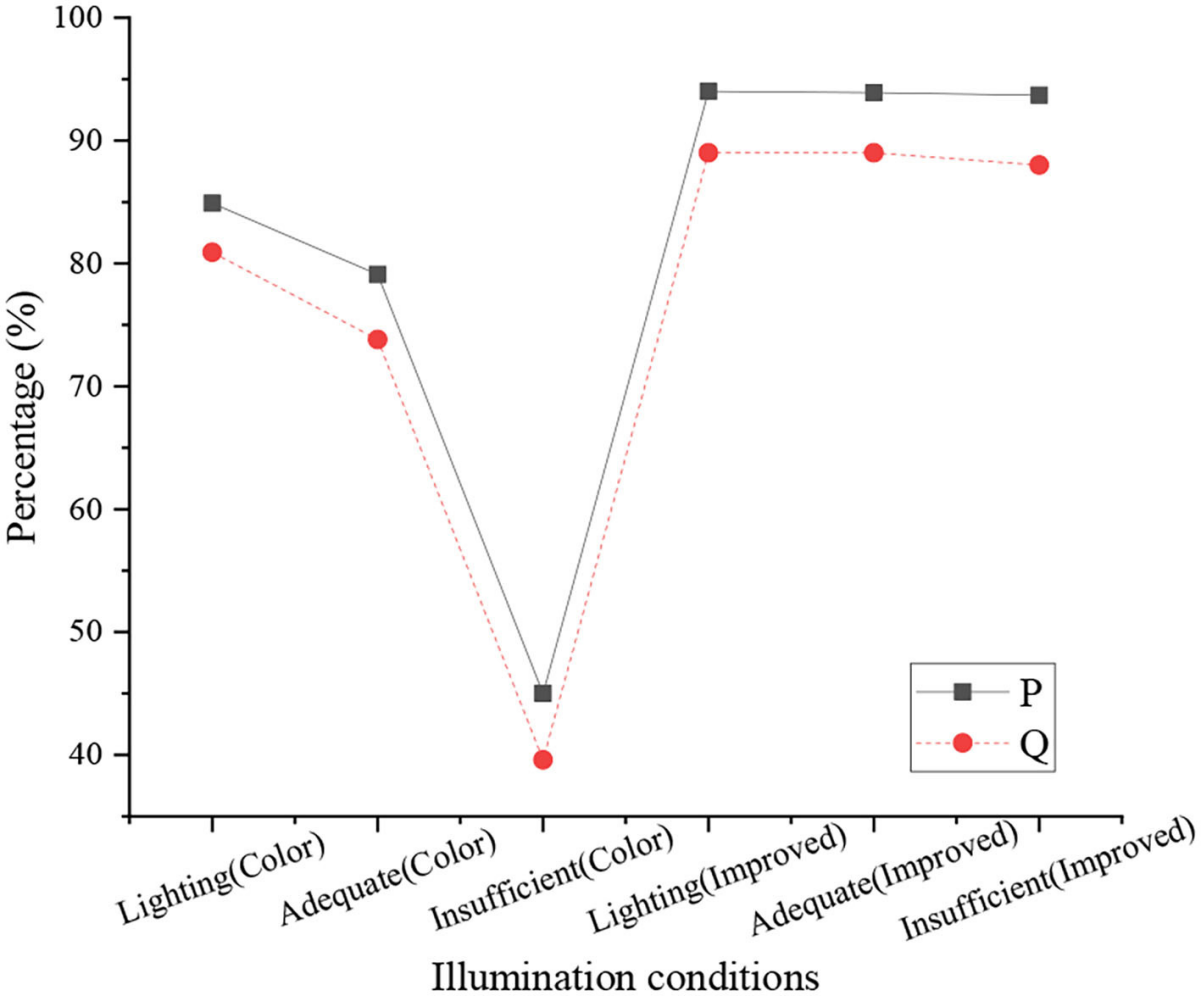
Performance comparison of color segmentation model and improved model.

The average pixel precious P and MIoU Q of the color segmentation model and the improved model under different lighting are shown in Figure 14.

Figure 14 indicates that the P of the improved model exceeds 90%, especially under insufficient natural light, the P of the improved model is 93.7%, while the average pixel accuracy of the color segmentation model under insufficient natural light is 45%. The Q of the improved model is about 89% in the three lighting modes, and the Q of the color segmentation model is larger in different lighting modes. It refers that the improved model algorithm has better stability.

**Figure 15:**
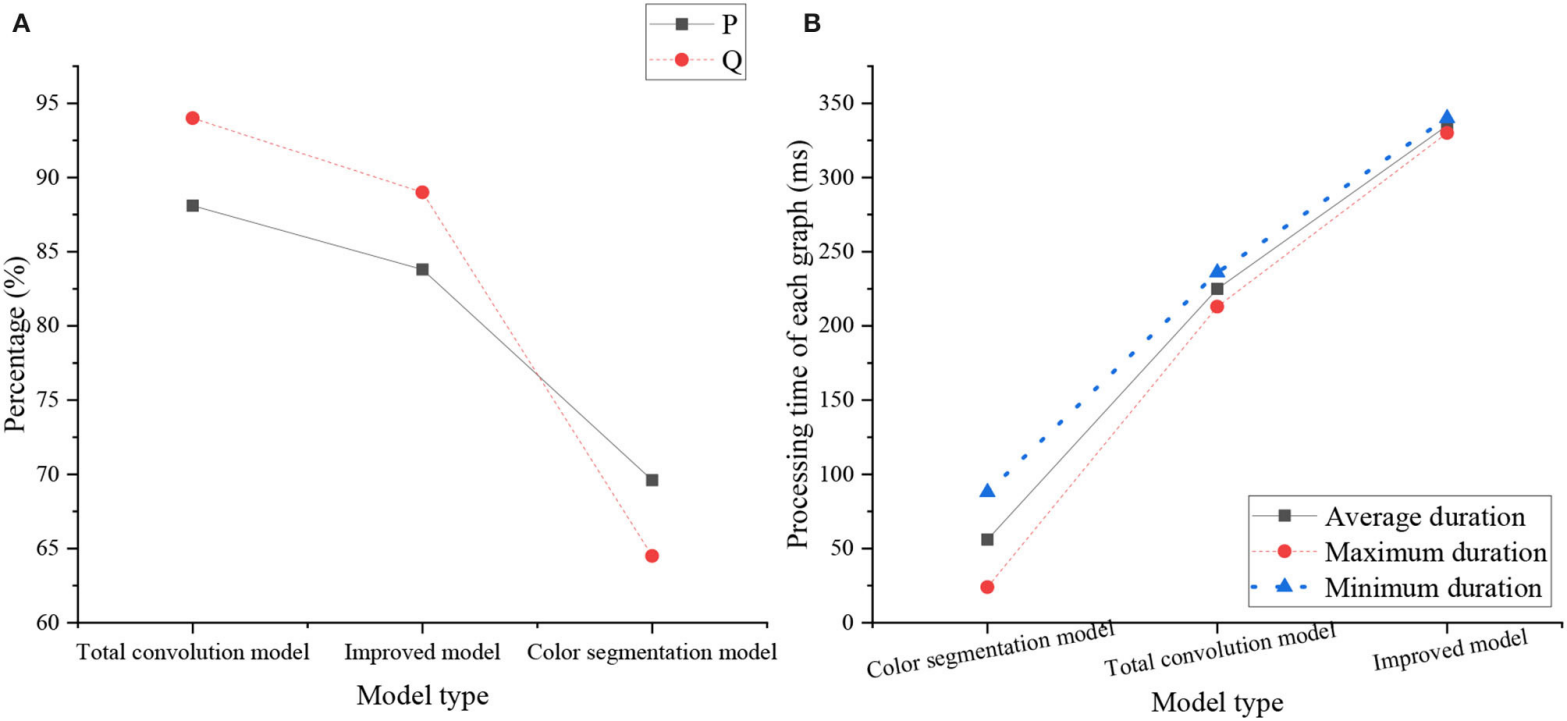
Performance comparison of the models [**(A)** The comparison between the FCN model and the improved model, **(B)** The comparison of running time of the three models].

The performance comparison of the color segmentation model, the FCN model, the improved model, and the comparison of the running time of the three models are shown in Figure 15.

Figure 15A shows that the P and Q of the improved model are higher than those of the FCN model. Due to the weak feature extraction ability of the FCN model, it is easy to be interfered by other nonsite line objects in the field line recognition, and the detection results are not accurate enough. In contrast, the improved model can complete the yield line detection more accurately due to its strong feature extraction ability. Figure 15B shows that the improved model takes the longest time due to the larger amount of computation, but the detection effect is good, the accuracy is high, and the detection speed basically meets the requirements of the competition.

## Conclusion

To improve the effectiveness of functional strength training for football players and the field detection stability of football robots, first the DL and human action recognition are combined under the background of AI to study intelligent training methods suitable for football players. Second, a functional strength training system is established by combining time flow and spatial flow networks. Based on CNN, an improved yield detection method is designed. Finally, the application ability of the system is evaluated. The results manifest that the intelligent training system is feasible, and the designed football robot has high field detection accuracy. The research provides a reference for the intelligent training of football players and the development of football robots. The training system will be mainly used for football training in schools and clubs to realize the autonomy and intelligence of football training and provide a certain reference for improving the overall level of football players in China. Field detection of football robots can be used in football robot games. But there are some drawbacks. There are few statistics about different actions in football in the dataset, and the main limitations are not to study the positioning of football robots. In the follow-up research, these two parts will be deeply analyzed to make the research content more comprehensive. The practical application value of research is that after further improvement, it will be expected to promote the level of football training in colleges and universities, and improve the level of football capabilities of the young players.

## Data Availability Statement

The raw data supporting the conclusions of this article will be made available by the authors, without undue reservation.

## Author Contributions

All authors listed have made a substantial, direct, and intellectual contribution to the work and approved it for publication.

## Funding

This work was supported by a grant from the Foundation of Social Science and Humanity, China Ministry of Education (Grant No. 19YJC890050), the Natural Science Foundation of Zhejiang Province of China (Grant No. LY18C110002), the Major Planning Program of Hangzhou Philosophy and Social Science Key Research Base (2021; Grant No. 2021JD19), and by the Foundation of Educational Commission of Zhejiang Province (Grant No. Y202147781).

## Conflict of Interest

The authors declare that the research was conducted in the absence of any commercial or financial relationships that could be construed as a potential conflict of interest.

## Publisher's Note

All claims expressed in this article are solely those of the authors and do not necessarily represent those of their affiliated organizations, or those of the publisher, the editors and the reviewers. Any product that may be evaluated in this article, or claim that may be made by its manufacturer, is not guaranteed or endorsed by the publisher.

## References

[B1] Abdelbaky A. Aly S. (2021). Human action recognition using three orthogonal planes with unsupervised deep convolutional neural network. Multimed. Tools Appl. 80, 20019–20043. 10.1007/s11042-021-10636-2

[B2] Antonioni E. Suriani V. Riccio F. Nardi D. (2021). Game strategies for physical robot soccer players: a survey. IEEE Trans. Games 13, 342–357. 10.1109/TG.2021.3075065

[B3] Aslam S. Herodotou H. Mohsin S. M. Javaid N. Ashraf N. Aslam S. (2021). A survey on deep learning methods for power load and renewable energy forecasting in smart microgrids. Renew. Sust. Energ. Rev. 144, 110992. 10.1016/j.rser.2021.110992

[B4] Bueno C. A. de Araujo Ribeiro-Alvares J. B. dos Santos Oliveira G. Grazioli R. Veeck F. Pinto R. S. (2021). Post-match recovery of eccentric knee flexor strength in male professional football players. Phys. Ther. Sport. 47, 140–146. 10.1016/j.ptsp.2020.11.032 33279801

[B5] Chen D. (2020). Fuzzy obstacle avoidance optimization of soccer robot based on an improved genetic algorithm. J. Ambient Intell. Human. Comput. 11, 6187–6198. 10.1007/s12652-019-01636-0

[B6] Chen D. Wawrzynski P. Lv V. (2021). Cyber security in smart cities: a review of deep learning-based applications and case studies. Sustain. Cities Soc. 66, 102655. 10.1016/j.scs.2020.102655

[B7] Chen X. Gao P. (2020). Path planning and control of soccer robot based on genetic algorithm. J. Ambient Intell. Human. Comput. 11, 6177–6186. 10.1007/s12652-019-01635-1

[B8] Colombini E. L. Drews-Jr P. L. Gonçalves L. M. (2022). Editorial notes for topical collection on robotica 2019. J. Intell. Robot. Syst. 104, 1–3. 10.1007/s10846-021-01557-1

[B9] Cournoyer J. Karton C. Koncan D. Gilchrist M. D. Cantu R. C. Hoshizaki T. B. (2021). Brain trauma exposure for American tackle football players 5 to 9 and 9 to 14 years of age. J. Biomech. 127, 110689. 10.1016/j.jbiomech.2021.110689 34416530

[B10] Elharrouss O. Almaadeed N. Al-Maadeed S. Bouridane A. Beghdadi A. (2020). A combined multiple action recognition and summarization for surveillance video sequences. Appl. Intell. 51, 690–712. 10.1007/s10489-020-01823-z

[B11] Fan L. Zhang T. Du W. (2020). Optical-flow-based framework to boost video object detection performance with object enhancement. Expert. Syst. Appl. 170, 114544. 10.1016/j.eswa.2020.114544

[B12] Gong J. Li R. Yao H. Kang X. Li S. (2019). Recognizing human daily activity using social media sensors and deep learning. Int. J. Env. Res. Public Health 16, 3955. 10.3390/ijerph16203955 31627356 PMC6843133

[B13] Guan S. Wang X. (2021). Optimization analysis of football match prediction model based on neural network. Neural Comput. Appl. 41, 1–17. 10.1007/s00521-021-05930-x

[B14] Haldorai A. Ramu A. (2020). Canonical correlation analysis based hyper basis feedforward neural network classification for urban sustainability. Neural Process. Lett. 53, 2385–2401. 10.1007/s11063-020-10327-3

[B15] Hong C. Jeong I. Vecchietti L. F. Kim J. (2021). AI world cup: robot-soccer-based competitions. IEEE Trans. Games. 13, 330–341. 10.1109/TG.2021.3065410

[B16] Houtman W. Martinez C. L. Wang S. Ketels A. Bruyninckx H. P. van de Molengraft M. J. G. (2021). Dynamic control of steerable wheeled mobile platforms applied to an eight-wheeled RoboCup Middle Size League soccer robot. Mechatronics 80, 102693. 10.1016/j.mechatronics.2021.102693

[B17] Jeong J. Yang J. Baltes J. (2022). Robot magic show as testbed for humanoid robot interaction. Entertain. Comput. 40, 100456. 10.1016/j.entcom.2021.100456

[B18] Khan M. A. Zhang Y. D. Khan S. A. Attique M. Rehman A. Seo S. (2020). A resource conscious human action recognition framework using 26-layered deep convolutional neural network. Multimed. Tools Appl. 80, 8. 35827–35849. 10.1007/s11042-020-09408-1

[B19] Khan S. Khan M. A. Alhaisoni M. Tariq U. Yong H. S. Armghan A.. (2021). Human action recognition: a paradigm of best deep learning features selection and serial based extended fusion. Sensors 21, 37941. 10.3390/s21237941 34883944 PMC8659437

[B20] Kuwana R. Ariji Y. Fukuda M. Kise Y. Nozawa M. Kuwada C.. (2020). Performance of deep learning object detection technology in the detection and diagnosis of maxillary sinus lesions on panoramic radiographs. Dentomaxillofac. Rad. 50, 20200171. 10.1259/dmfr.20200171 32618480 PMC7780831

[B21] Lagemann C. Lagemann K. Mukherjee S. Schröder W. (2021). Deep recurrent optical flow learning for particle image velocimetry data. Nat. Mach. Intell. 3, 641–651. 10.1038/s42256-021-00369-0

[B22] Materne O. Chamari K. Farooq A. Weir A. Hölmich P. Bahr R. (2020). Injury incidence and burden in a youth elite football academy: a four-season prospective study of 551 players aged from under 9 to under 19 years. Br. J. Sport. Med. 55, 493–500. 10.1136/bjsports-2020-102859 33199359

[B23] Newman J. Lin J. W. Lee D. J. Liu J. J. (2021). Automatic annotation of American Football Video footage for game strategy analysis. J Electron. Imag. 21, 303. 10.2352/ISSN.2470-1173.2021.6.IRIACV-303

[B24] Ozcan T. Basturk A. (2020). Performance improvement of pre-trained convolutional neural networks for action recognition. Comput. J. 64, 1. 1715–1730. 10.1093/comjnl/bxaa029

[B25] Pareek P. Thakkar A. (2020). A survey on video-based human action recognition: recent updates, datasets, challenges, and applications. Artif. Intell. Rev. 54, 2259–2322. 10.1007/s10462-020-09904-8

[B26] Parim C. Güneş M. S. Büyüklü A. H. Yildiz D. (2021). Prediction of match outcomes with multivariate statistical methods for the group stage in the UEFA Champions League. J. Hum. Kinet. 79, 197–209. 10.2478/hukin-2021-0072 34400999 PMC8336563

[B27] Park C. Kim B. Kim Y. Eum Y. Song H. Yoon D. (2022). Carved turn control with gate vision recognition of a humanoid robot for giant slalom skiing on ski slopes. Sensors 22, 816. 10.3390/s22030816 35161561 PMC8838643

[B28] Pons E. Ponce-Bordón J. C. Díaz-García J. López del Campo R. Resta R. Peirau X.. (2021). A longitudinal exploration of match running performance during a football match in the Spanish La Liga: a four-season study. Int. J. Env. Res. Public Health 18, 1133. 10.3390/ijerph18031133 33525322 PMC7908616

[B29] Pratama A. T. (2021). Augmented reality trasnportasi darat menggunakan FAST Corner Detection dan Lucas Kanade. JATISI 8, 1663–1671. 10.35957/jatisi.v8i3.1076

[B30] Sarker H. (2021). Deep learning: a comprehensive overview on techniques, taxonomy, applications and research directions. SN Comput. Sci. 2, 1–20. 10.1007/s42979-021-00815-1 34426802 PMC8372231

[B31] Setyawan N. Mardiyah N. A. Zulfatman Z. Fajar D. N. (2022). Navigasi Robot Sepak Bola Beroda Menggunakan particle filter localization. Cyclotron 5, 116. 10.30651/cl.v5i1.9419

[B32] Shen C. Luong T. Ho J. Djailani I. (2019). Social media marketing of IT service companies: analysis using a concept-linking mining approach. Ind. Market. Manag. 90, 593–604. 10.1016/j.indmarman.2019.11.014

[B33] Somu N. Gauthama Rama M. R. Ramamirtham K. (2021). A deep learning framework for building energy consumption forecast. Renew. Sust. Energ. Rev. 137, 110591. 10.1016/j.rser.2020.11059132143371

[B34] Stoeve M. Schuldhaus D. Gamp A. Eskofier B. M. (2021). From the laboratory to the field: IMU-based shot and pass detection in football training and game scenarios using deep learning. Sensors 21, 3071. 10.3390/s21093071 33924985 PMC8124919

[B35] Suwarno I. Ma'arif A. Raharja N. M. Shomad M. A. (2022). Using a combination of PID control and Kalman filter to design of IoT-based telepresence self-balancing robots during COVID-19 pandemic. Emerg. Sci. J. 4, 241–261. 10.28991/esj-2021-SP1-016

[B36] Teixeira J. E. Forte P. Ferraz R. Leal M. Ribeiro J. Silva A. J. (2021). Monitoring accumulated training and match load in football: a systematic review. Int. J. Env. Res. Pub. He. 18, 3906. 10.3390/ijerph18083906 33917802 PMC8068156

[B37] Thakkar P. Shah M. (2021). An assessment of football through the lens of data science. Ann. Data Sci. 71, 1–14. 10.1007/s40745-021-00323-2

[B38] Ullah A. Muhammad K. Hussain T. Baik S. W. (2020). Conflux LSTMs network: a novel approach for multi-view action recognition. Neurocomputing 435, 321–329. 10.1016/j.neucom.2019.12.151

[B39] Vella A. Clarke A. C. Kempton T. Ryan S. Holden J. Coutts A. J. (2021). Possession chain factors influence movement demands in elite Australian football match-play. Sci. Med. Football 5, 72–78. 10.1080/24733938.2020.1795235 35073233

[B40] Wang T. (2022). Exploring intelligent image recognition technology of football robot using omnidirectional vision of internet of things. J Supercomput. 15, 1–20. 10.1007/s11227-022-04314-9

[B41] Wang W. G. Shen J. B. Xie J. W. Cheng M. M. Ling H. Borji A. (2019). Revisiting video saliency prediction in the deep learning era. IEEE T. Pattern Anal. 43, 220–237. 10.1109/TPAMI.2019.2924417 31247542

[B42] Watanabe K. Ma Y. Kono H. Suzuki H. (2022). A self-localization method using a genetic algorithm considered kidnapped problem. J. Adv. Comput. Intell. Intell. Inform. 26, 32–41. 10.20965/jaciii.2022.p0032

[B43] Zhang D. Zhan J. Tan L. Gao Y. Župan R. (2020). Comparison of two deep learning methods for ship target recognition with optical remotely sensed data. Neural Comput. Appl. 33, 4639–4649. 10.1007/s00521-020-05307-6

[B44] Zhao D. Wu Y. Wang C. Shen C. Tang J. Liu J. (2021). Gray consistency optical flow algorithm based on mask-R-CNN and a spatial filter for velocity calculation. Appl. Optics 60, 10600–10609. 10.1364/AO.441233 35200922

